# Gender differences in modifiable risk factors for hip fracture: 10-year follow-up of a prospective study of 0.5 million Chinese adults

**DOI:** 10.1111/joim.13429

**Published:** 2021-12-22

**Authors:** Pang Yao, Sarah Parish, Derrick A Bennett, Huaidong Du, Ling Yang, Yiping Chen, Yu Guo, Canqing Yu, Gang Zhou, Jun Lv, Liming Li, Zhengming Chen, Robert Clarke

**Affiliations:** 1 Clinical Trial Service Unit and Epidemiological Studies Unit (CTSU), Nuffield Department of Population Health, University of Oxford, UK; 2 NIHR Oxford Biomedical Research Centre, Oxford, UK; 3 Medical Research Council Population Health Research Unit at the University of Oxford, Oxford, UK; 4 Fuwai Hospital Chinese Academy of Medical Sciences, National Center for Cardiovascular Diseases, Beijing, China; 5 Department of Epidemiology, School of Public Health, Peking University Health Science Center, Beijing, China, Peking University Center for Public Health and Epidemic Preparedness & Response, Beijing, China; 6 NCDs Prevention and Control Department, Henan CDC

**Keywords:** hip fracture, incidence rate, risk factors, population attributable fractions

## Abstract

**Importance:**

Reliable evidence on modifiable risk factors for hip and major osteoporotic fractures in untreated populations could inform prevention strategies for hip fracture worldwide.

**Objectives:**

To estimate the age- and sex-specific incidence of hip, major osteoporotic and any fractures and examine their associated risk factors in Chinese adults.

**Design, Setting, and Participants:**

Prospective study of 512,715 adults, aged 30-79 years, recruited for the China Kadoorie Biobank from 10 diverse areas in China in 2004-2008 and followed up for 10 years.

**Exposures:**

Sociodemographic factors (e.g. age, sex, and education), medical history (e.g. CVD, diabetes, fracture, and rheumatoid arthritis), anthropometry (e.g. weight, waist-hip ratio) and lifestyle factors (e.g. smoking, alcohol drinking, physical activity, diet).

**Main Outcomes and Measures:**

Age- and sex-specific incidence rates of fracture types, and adjusted hazard ratios (HR) and population attributable fractions (PAF) for individual risk factors associated with fracture types.

**Results:**

The incidence rates of hip fracture in Chinese adults were 5.1 (95%CI 5.0-5.3) per 10,000 person-years (2616 cases); and were higher in women than men (5.8 [5.5-6.1] vs 4.2 [3.9-4.5]) and increased by about 2 to 3-fold per 10 years older age. Among men, five risk factors for hip fracture including low education (HR=1.23; 95%CI 1.04-1.45), regular smoker (1.22, 1.03-1.45), lower weight (lowest vs other quintiles: 1.59, 1.34-1.88), alcohol drinker (1.18, 1.02-1.36), and prior fracture (1.62, 1.33-1.98) accounted for 44.3% of hip fractures. Among women, five risk factors including lower weight (lowest vs other quintiles: 1.30, 1.15-1.46), low physical activity (lowest vs other quintiles: 1.22, 1.10-1.35), diabetes (1.62, 1.41-1.86), prior fracture (1.54, 1.33-1.77), and self-rated poor health (1.29, 1.13-1.47), accounted for 24.9% of hip fractures. Associations of these risk factors with major osteoporotic (6857 cases) or any (15762 cases) fractures were weaker than those with hip fracture.

**Conclusions:**

The age- and sex-specific incidence rates of hip fracture in Chinese adults were comparable with those in Western populations. Five potentially modifiable factors accounted for half of all hip fractures in men and one quarter in women.

## Introduction

Osteoporosis is characterized by low bone mass and micro-architectural deterioration of bone tissue, resulting in bone fragility and susceptibility to fracture.^1^ Approximately 1 in 2 women and 1 in 5 men aged 50 years or older will experience an osteoporotic fracture in their remaining lifetime.^2–4^ Hip fractures are the most serious type of osteoporotic fractures, with an approximate 30% absolute risk of death in the year following hip fracture.^5^ Fractures of vertebrae, humerus and forearm or hip are referred to as “major osteoporotic fractures”.^6^ Both hip and major osteoporotic fractures are more common in women than in men and the incidence of both increases exponentially with age.^7,8^

Previous studies reported lower incidence rates of hip fractures in Asian than in Western populations, with reported age-standardized incidence rates per 10,000 person-years of 14 in China, 13 in India, 44 in Denmark, 42 in Norway, 40 in Sweden and 25 in UK.^9,10^ The global burden of hip fractures is increasing worldwide, and current estimates suggest that the annual incidence will reach 4.5 million cases per year by 2050. About half of these cases are likely to occur in Asia, particularly in China.^11,12^ Previous city-level studies of hospitalised cases have reported differences in the age- and sex-specific incidence rates of hip fractures between different areas of China, albeit the reasons for such differences are uncertain.^10,13–16^

Several risk factors have been consistently associated with higher risks of hip fractures, including age, sex, weight, height and prior history of fracture, but the relevance of diabetes, alcohol use or socioeconomic status is uncertain.^17,18^ While gender differences in incidence and mortality of hip fracture have been consistently reported,^19^ many previous studies of hip fracture have focused exclusively in women^17,20–24^ or on a single set of risk factors,^21,24,25^ and little is known about the relative importance major modifiable risk factors in low and middle-income countries (LMICs), such as China.

The present report examined the incidence of hip and major osteoporotic fractures and their associated risk factors in a 10-year follow-up of a cohort study of >0.5 million adults recruited from 10 diverse areas in China.^26^ The aims of this report were to: (i) estimate the age- and sex-specific incidence rates of fracture types (i.e., hip fracture, major osteoporotic fracture and any fracture), overall and by areas within China; and (ii) identify major risk factors for fracture types; and (iii) assess the population attributable fractions (PAF) for any potentially modifiable risk factors for hip fracture.

## Methods

### Study population

Details of the design and methods used for recruitment for the China Kadoorie Biobank (CKB) study have been previously reported.^26^ Overall, a total of 512,715 aged 30-79 years participated in the baseline survey between June 2004 and July 2008. Local, national and international ethics approvals were obtained, and all participants provided written informed consent.

### Data collection

At the local study assessment clinics, participants completed an interviewer-administered laptop-based questionnaire that included questions on socio-demographic characteristics, smoking, alcohol consumption, diet, physical activity, personal and family medical history and current use of medication. Physical measurements were recorded using calibrated instruments for height, weight, hip and waist circumference, bioimpedance, lung function, blood pressure and heart rate (see eTable 1 for details).

### Follow-up for incident cases of fracture types

The vital status of each participant was determined periodically through China’s Disease Surveillance Points (DSP) system and national health insurance systems, supplemented by annual active confirmation through street committees or village administrators.^26^ Data on the incidence of major diseases and any hospitalisations were collected by linkage, using each participant’s unique national identification number, with disease registries and national health insurance claims databases and all mortality registries. All deaths or hospital admissions were coded using the International Classification of Diseases, 10^th^ Revision (ICD-10) by trained DSP staff who were blinded to other information collected in the study. By 1 Jan 2017, 44066 (8.6%) participants died and 4751 (0.9%) were lost to follow-up.

The primary outcomes were admission to hospital with hip fracture, and the secondary outcomes included major osteoporotic fracture, any fracture and osteoporosis (see eAppendix for ICD-10 codes used to define disease endpoints).^27^ All analyses were restricted to known first-ever hospitalisation events for that outcome during follow-up period.

### Statistical analyses

All the analyses were performed separately for men and women. The incidence rates of fracture types were standardized by age and study area, with exposure time (years) calculated from date of enrolment until the incident fracture, death, or censoring date (December 31, 2016) for follow-up. The incidence rates and their 95% confidence intervals (95% CI) were estimated using the number of fracture cases per 10,000 person-years (py) and the SEs were estimated assuming the number of cases had a Poisson distribution.^28^ Cox Proportional Hazards models stratified by area were used to estimate the sex-specific hazard ratios (HR) for fracture types associated with individual risk factors, in univariable and multivariable analyses (see eAppendix). Population attributable fractions (PAFs, expressed as a percentage) assuming a causal relationship were estimated for potentially modifiable risk factors (i.e., excluding age and height), separately in men and women. ^29–31^ Details of the methodology used to estimate PAFs are provided in eTable 1.^32^ Collinearity between multiple risk factors was assessed using variance inflation factor (VIF), and a VIF factor >10 was used to indicate collinearity. In sensitivity analyses, we excluded individuals who reported a prior history of any fracture at baseline and fractures occurring during the first 5 years of follow-up. All analyses were conducted using R version 3.6.2.

### Results

Among the 512,715 participants included, the mean age was 52 years and 59% were women. Compared with men, women were younger, less educated, had lower household income, and much less likely to smoke (3.3% vs 74.2%) and drink alcohol (2.5% vs 37.0%). In contrast, women had a higher prevalence of overweight or obesity (45.3 vs 41.9%). The prevalence of a prior history of any fracture at enrolment was 8.8% in men and 5.7% in women, respectively (Table 1).

During a median follow-up of 10 years, a total of 15,762 participants were hospitalised for the first time with any fracture (2616 with hip fracture and 6857 with major osteoporotic fracture) and 2690 had a reported diagnosis of osteoporosis. The overall incidence rates of hip fracture per 10,000 py were 5.1 (95% CI 5.0-5.3) with higher rates in women than men (5.8 [5.5-6.1] vs 4.2 [3.9-4.5]), and in rural than urban areas (5.5 [5.2-5.8] vs 4.7 [4.5-5.0]) (Table 2). Across the 10 study areas, the age-adjusted incidence rates of hip fracture varied by almost 5-fold in men and 10-fold in women (eFigure 1). Similar, albeit less extreme, associations were observed for major osteoporotic fracture, any fracture and osteoporosis (Table 2).

**Figure 1:**
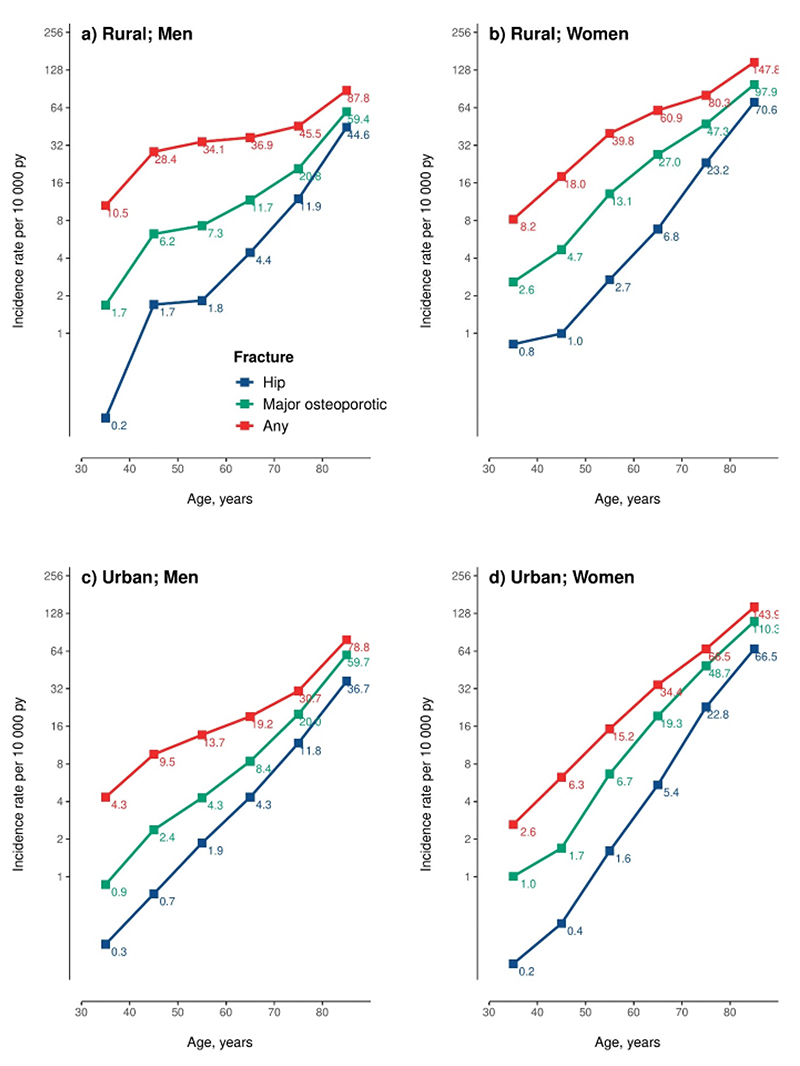
Age- and sex-specific incidence rates of different fracture types, by area The age-specific incidence rates of different fracture types were estimated separated in (a) rural men, (b) rural women, (c) urban men and (d) urban women. The numbers next to the squares are the incidence rates per 10,000 person-years (pys).

The incidence rates of hip fracture were slightly higher in men than in women up until age 50 years, after which incidence rates increased much more rapidly in women than in men, increasing from 1.6 at 50-59 years to 66.5 per 10,000 py at 80 years or older in urban women and from 2.7 to 70.6 per 10,000 py in rural women (Figure 1). Among men, the corresponding age-related changes were much less extreme in both urban (varied from1.9 to 36.7) and rural areas (from 1.8 to 44.6) (Figure 1). Similarly, the proportion of hip fracture to any fracture increased rapidly with age, from 2-9% at age 30-39 years to 46-51% at age 80 years or older.

**Figure 2:**
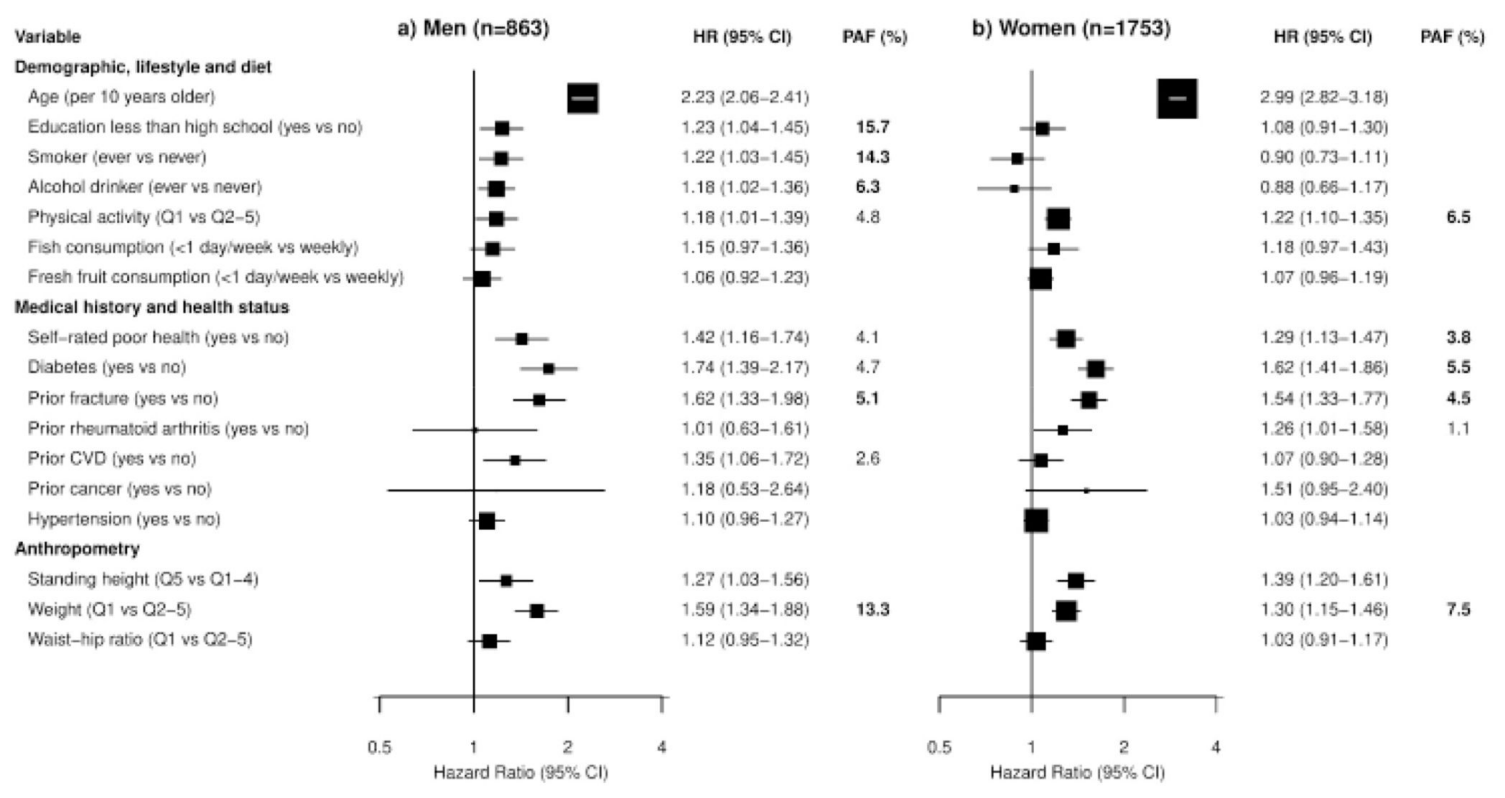
Associations of selected major risk factors with hip fracture in men and women Multivariable analyses after stratifying by area were used to explore the potential risk factors separately in men and women. The size of the squares is proportional to the inverse of the variance of the log HRs. Population attributable fractions for hip fracture are presented for the modifiable risk factors. Those PAF values highlighted in bold are the top 5 risk factors in men and women, respectively. Abbreviations: Q, quintile.

Several major risk factors (e.g., low education, low physical activity, low consumption of fish or fresh fruit, and history of fracture) were also independently associated with risk of hip fracture in univariable analyses in men and women (eTable 2). In multivariable analyses (Figure 2), age was more strongly associated with higher risks of hip fracture in women than men (per 10 years older: 2.99 [2.82-3.18] vs 2.23 [2.06-2.41]; *χ^2^* =31.3, *P*=2.2e-08). Physical activity, self-rated poor health, diabetes and history of fracture were each strongly associated with higher risks of hip fracture in both men and women (HR range: 1.18-1.74). Regular smokers or regular alcohol drinkers were each associated with higher risks of hip fracture in men, but not in women. Lower levels of education and prior CVD were also associated with higher risks of hip fracture, but only in men. Prior rheumatoid arthritis was associated with a higher risk of hip fracture only in women.

Taller standing height was positively associated with a higher risk of hip fracture in both men and women (eFigure 2). The association of height with hip fracture was log-linear, with each 1 SD taller height associated with an adjusted HR of 1.07 (0.99-1.16) in men and 1.16 (1.10-1.23) in women. In contrast, all measures of adiposity, including weight, hip circumference, waist circumference, waist-hip ratio, waist-height ratio and BMI were each inversely associated with risk of hip fracture (eFigure 2). Likewise, physical activity was inversely associated with hip fracture in both men and women (eFigure 3). Height, weight and waist-hip ratio were selected for multivariable analyses (as these were less correlated with each other, with correlation coefficients <0.6), with the risk group being the top quintile for height, and bottom quintile for weight, waist-hip ratio and physical activity, respectively. In multivariable analyses, taller standing height and lower weight were each strongly associated with higher risks of hip fracture in both men and women (Figure 2). The associations of these risk factors with major osteoporotic fracture and any fracture were similar, albeit less extreme, as those with hip fracture (eFigure 4). Likewise, age, self-rated poor health, history of fracture or rheumatoid arthritis were also associated with higher risks of osteoporosis in men or women (eFigure 4). There was no evidence of collinearity between any of these risk factors and risk of hip fracture.

Figure 2 shows that the modifiable risk factors which accounted for highest proportions of the PAF for hip fracture differed between men and women, respectively. Low education was the most important risk factor for hip fracture in men, accounting for 15.7% of the PAF, followed by regular smoker (14.3%), lower weight (13.3%), alcohol drinker (6.3%), prior fracture (5.1%), low physical activity (4.8%), diabetes (4.7%), self-rated poor health (4.1%) and prior CVD (2.6%). Together the top five modifiable factors based on the PAF accounted for 44.3% of all hip fractures in men. In women, lower weight accounted for 7.5% of hip fracture, followed by low physical activity (6.5%), diabetes (5.5%), prior fracture (4.5%), self-rated poor health (3.8%) and prior rheumatoid arthritis (1.1%). Together the top five modifiable factors accounted for 24.9% of all hip fractures in women.

Table 3 shows the distribution of the top five modifiable risk factors by 10 areas separately for men and women. Compared to the regional differences in the age-adjusted incidence rates of hip fracture (~5 fold in men and ~10 fold in women), the difference decreased to ~4 fold in both men and women when further adjusted for the leading five risk factors. The associations of these risk factors with hip fracture were largely unaltered by exclusion of participants with a prior history of fracture at baseline (eFigure 5), or by exclusion of fractures occurring during the first 5 years of follow-up (eFigure 6), or both (eFigure 7).

## Discussion

This study demonstrated that the incidence rates of hip fracture in Chinese adults were comparable with those in European populations,^5^ and were higher in women than in men at age 50 years or older. The incidence rates for hip fracture at age ≥80 years were 40.3 in men and 68.2 per 10,000 py in women, while in the UK, the corresponding rates obtained from primary care records were 40.1 and 89.4, respectively.^7^ Overall, five potentially modifiable risk factors accounted for about half of all hip fractures in men and a quarter in women. Several modifiable markers of frailty (low weight and low physical activity) and adverse lifestyle factors (smoking, alcohol, low education particularly in men) and medical history (diabetes, prior fracture, or rheumatoid arthritis) accounted for most hip fractures.

The incidence of hip fracture varied by 5 to 10-fold between different areas in China, which largely reflected differences between levels of potentially modifiable risk factors across these areas in men women. However, it was not possible to fully exclude the possibility of differences in health systems may account for some of these geographic differences. The China Health and Retirement Longitudinal Study also reported that the incidence of hip fracture was higher in Zhejiang, Sichuan and Guangxi provinces than other areas in China.^33^

Previous studies of hip fracture were limited to city-level studies of hospitalised cases that were also constrained by small sample size, short duration of follow-up, and covered limited geographic areas within China.^10,13–16^ One prospective study reported higher incidence rates of hip fracture among urban adults (age ≥55 years, 9.9-12.2 and 15.6-20.4 per 10,000 py in men and women, respectively) than the CKB study (5.5 and 8.2 per 10,000 py),^34^ but the mean age of the latter study participants was considerably older than in the present study (77.1 vs 60.0 years). A systematic review of the worldwide incidence of hip fracture reported lower incidence rates of hip fracture in Chinese than in Western populations.^9^ Several studies have reported stabilized or declining incidence rates of hip fracture in North America,^8,28,35^ Europe,^36^ and in Hong Kong and Singapore (two economically advanced cities in Asia),^37^ but rates of hip fracture in LMICs, such as China, appear to be increasing.^10,38^ Both the increasing incidence rates of fracture and higher proportions of the population that survive to old age would be expected to increase and highlight the need for more effective strategies for prevention of fractures.

In contrast with previous studies in China, which mainly used cross-sectional or case-control study designs,^18,25,33^ or restricted to Chinese postmenopausal women,^21^ the present study provided a detailed evaluation of potentially modifiable risk factors for hip fracture in Chinese men and women independently. Consistent with current fracture risk assessment tools,^39^ increasing age, taller height, lower weight, and prior diabetes or fracture were independent risk factors for hip fractures in both men and women. The association of height with risk of hip fracture observed in the present study probably reflects biomechanical mechanisms.^40^ The length of the femur is a determinant of the fracture risk after a fall, and individuals with taller height require less force to sustain a fracture. Individuals with higher levels of adiposity are believed to have more optimum physical protection of a higher mass of gluteofemoral adipose tissue, which reduces the impact of falling and subsequent risk of fracture.^41^ Low weight, a marker of frailty, is a risk factor for hip fracture and this effect may be mediated through low BMD, as decreased muscle mass and strain may decrease BMD and structural integrity of the underlying bones.^41^

Low levels of education were associated with the highest population attributable fraction of the risk factors in men. An inverse association between highest level of educational attainment and risk of hip fracture was previously reported in some studies in Europe and USA,^42^ and in China^33^ but not in others.^41^ It is possible that people in lower education attainment may have more unhealthy lifestyles and are less likely to undergo screening for bone and other diseases, and suffer higher risks of falls.

Low physical activity has also been associated with higher risks of different fracture types in several previous studies, which may reflect the effects of frailty.^24^ Higher levels of physical activity may reduce the risks of hip fracture by improving balance, coordination, and muscle strength, but participation in physical activity may also increase the risks of falls, injury and other fractures.^23,24,43^ Other studies ^23,24^ have also reported that higher levels of physical activity were associated with lower risks of hip fracture, but higher risks of knee, elbow, ankle or wrist fracture.^23,24^

Previous studies in Western populations have also demonstrated the importance of current cigarette smoker and alcohol drinker as independent risk factors for hip fractures in women^17,44^, in men^45,46^ or both.^18^ However, in CKB few Chinese women regularly smoked tobacco or drank alcohol. The strength of the associations for current smoking in men (HR 1.22) was similar to a previous study in Singapore (1.23 for men and 1.27 for women),^47^ but lower than previous studies in Western populations ^45, 46^ Nevertheless, approximately 15% of all hip fractures in the present study population were attributable to tobacco smoking, consistent with previous estimates of 19% in Western studies.^48^

The lack of association of hip fracture with RA may reflect the smaller number of men with RA. History of CVD was not associated with higher risk of hip fracture in the multivariable analyses, perhaps reflecting confounding by low levels of physical activity and diabetes. Consistent with previous reports in Western populations, individuals with a self-reported prior history of poor health in the present study had a higher risk of hip fracture in both men and women, possibly reflecting effects of frailty, due to underlying disease or treatment.^49^

The present study had several strengths, including prospective study design, large numbers of well-characterised participants enrolled from 10 diverse areas, and ability to assess incident cases of different fracture types. However, the study also had several limitations including not being representative of the Chinese population, but this does not preclude generalizability of the relative risks with individual risk factors.^50^ The available evidence collected on osteoporosis was limited to those identified during admission to hospital, rather than any systematic screening for osteoporosis. Importantly, many of the observed risk factor associations for hip and major osteoporotic fractures were concordant with those reported by previous studies in European populations. Public health strategies for prevention of hip and major osteoporotic fracture should target older people with selected markers of frailty (low weight and low physical activity) and presence of other potentially modifiable risk factors (smoking, alcohol, low education particularly in men) to reduce morbidity and mortality associated with hip and major osteoporotic fractures worldwide.

## Supplementary Material

supplementary

## Figures and Tables

**Table 1:** Selected baseline characteristics in men and women

Characteristics	Overall (512,715)	Men (210,205)	Women (302,510)
**Demographic factors**			
Age, years	52.0 (10.7)	52.9 (10.9)	51.5 (10.5)
Urban residents, %	44.1	43.3	44.9
Education less than high school, %	79.0	73.9	82.5
Household income (>20000 yuan/year), %	42.7	45.6	40.8
**Lifestyle factors**			
Regular smokers, %	32.4	74.2	3.3
Regular drinkers, %	16.7	37.0	2.5
Physical activity, MET h/day	21.1 (13.9)	22.4 (15.3)	20.2 (12.8)
**Dietary factors**			
Meat (≤3 days/week)	52.8	48.4	55.9
Fish (<1 day/week)	53.2	50.9	54.8
Fresh fruit (<1 day/week)	40.4	44.0	37.8
Dairy (non-consumers)	68.4	69.3	67.8
**Medical history and health status**, %			
Poor self-rated health	10.4	8.8	11.5
Diabetes*	5.9	5.5	6.3
History of fracture	6.9	8.8	5.7
History of rheumatoid arthritis	2.1	1.4	2.5
History of CVD†	4.5	4.8	4.3
History of cancer	0.5	0.4	0.5
Hypertension	33.5	35.8	31.9
**Anthropometry**			
Standing height, cm	158.9 (8.3)	165.3 (6.5)	154.1 (6.0)
Weight, kg	59.8 (10.8)	64.3 (10.9)	56.6 (9.5)
BMI, kg/m^2^	23.7 (3.4)	23.5 (3.2)	23.8 (3.5)
BMI≥24 kg/m^2^, %	43.8	41.9	45.3
Waist-hip ratio	0.88 (0.07)	0.90 (0.06)	0.87 (0.07)
SBP, mmHg	131 (21)	132 (20)	130 (22)

Mean (SD) and percentages were standardized by age at baseline (5-year intervals) and area of the CKB population.

Abbreviations: MET-h, metabolic equivalent of task-hours; SBP, systolic blood pressure;

* Includes those with a self-reported diagnosis by a doctor of diabetes and screen-detected cases at baseline.

† Includes self-reported diagnosis by a doctor of CHD, stroke or TIA (transient ischemic attack

**Table 2:** Standardized incidence rates (per 10,000 person-years) of different types of fracture and osteoporosis

	Hip fracture		Major osteoporotic fracture*		Any fracture		Osteoporosis	
	No. of events	Rate (95% CI)	No. of events	Rate (95% CI)	No. of events	Rate (95% CI)	No. of events	Rate (95% CI)
All	2616	5.1 (5.0-5.3)	6857	13.5 (13.2-13.9)	15762	31.4 (30.9-31.9)	2690	5.3 (5.1-5.5)
Sex								
Men	863	4.2 (3.9-4.5)	1898	9.3 (8.9-9.7)	5472	27.0 (26.3-27.7)	515	2.5 (2.3-2.7)
Women	1753	5.8 (5.5-6.1)	4959	16.4 (16.0-16.9)	10290	34.3 (33.7-35.0)	2175	7.2 (6.9-7.5)
Age at risk, years								
30-39	10	0.5 (0.2-0.8)	38	1.7 (1.2-2.4)	148	6.8 (5.8-8.0)	9	0.4 (0.2-0.8)
40-49	132	1.0 (0.8-1.1)	534	3.9 (3.6-4.3)	2170	16.0 (15.4-16.7)	161	1.2 (1.0-1.4)
50-59	331	2.1 (1.9-2.3)	1366	8.6 (8.1-9.0)	4339	27.4 (26.6-28.2)	497	3.1 (2.8-3.4)
60-69	664	5.5 (5.1-5.9)	2184	18.0 (17.3-18.8)	4881	40.8 (39.7-42.0)	892	7.3 (6.9-7.8)
70-79	1112	17.9 (16.9-19.0)	2185	35.5 (34.0-37.0)	3476	57.0 (55.1-58.9)	957	15.4 (14.5-16.4)
80+	367	55.4 (49.9-61.4)	550	84.3 (77.4-91.6)	748	116.5 (108.3-125.2)	174	26.2 (22.4-30.4)
Area								
Rural	1413	5.5 (5.2-5.8)	4052	15.2 (14.7-15.6)	10757	39.6 (38.9-40.4)	1860	7.0 (6.7-7.4)
Urban	1203	4.7 (4.5-5.0)	2805	11.4 (10.9-11.8)	5005	21.0 (20.4-21.6)	830	3.3 (3.0-3.5)

Standardized by age (10-year intervals), sex, and study area (10 areas) of CKB population (where appropriate)

* Includes fractures of hip, vertebra, humerus and ulna/radius.

**Table 3:** Regional differences in incidence rates of hip fracture and their associated risk factors (top 5) in men and women

Risk factors	Zhejiang	Liuzhou	Hunan	Sichuan	Suzhou	Gansu	Harbin	Haikou	Qingdao	Henan
*Men*										
No. of participants	24027	19321	26370	21315	22363	19298	23252	10794	15624	27841
**Age-adjusted rate†**	**6.3**	**5.3**	**3.8**	**3.8**	**3.6**	**3.3**	**2.9**	**2.5**	**2.1**	**1.3**
Education less than high school	94.2	55.3	88.4	89.4	86.5	83.6	40.1	49.9	58.8	78.5
Ever-regular smokers, %	82.3	65.3	78.1	78.5	81.6	78.4	68.6	52.6	74.0	71.9
Weight Q1, %	24.1	14.9	37.5	35.4	14.9	27.9	6.0	19.6	3.0	13.4
Ever-regular drinkers, %	43.1	29.9	31.6	56.9	45.6	9.2	51.8	18.2	50.4	26.5
Prior fracture, %	11.3	12.1	8.1	5.4	18.4	3.9	6.9	4.0	3.8	9.6
**Multivariable-adjusted rate‡**	**5.7**	**6.0**	**3.5**	**3.4**	**3.4**	**3.4**	**3.6**	**3.0**	**2.4**	**1.5**
*Women*										
No. of participants	33677	30852	33530	34371	30896	30589	34304	18892	19884	35515
**Age-adjusted rate†**	**9.6**	**6.1**	**5.4**	**4.6**	**4.9**	**3.7**	**3.6**	**3.1**	**3.4**	**1.0**
Weight Q1, %	23.3	18.0	34.0	32.0	16.6	22.5	9.1	25.4	3.1	12.1
Physical activity Q1, %	9.7	24.9	24.1	9.3	15.8	8.7	29.1	32.8	33.3	23.8
Diabetes, %*	5.8	8.3	4.1	4.0	5.3	3.6	9.6	6.6	10.4	5.7
Prior fracture, %	8.7	9.1	4.6	2.5	15.0	1.3	4.4	2.4	1.9	4.0
Self-rated poor health, %	5.1	10.5	7.0	22.9	12.3	12.4	11.5	7.3	5.3	14.8
**Multivariable-adjusted rate‡**	**7.8**	**8.1**	**4.7**	**4.6**	**4.7**	**4.6**	**4.8**	**4.1**	**3.3**	**2.0**

Areas are ordered by incidence rates of hip fractures in men. Comparisons of numbers (percentages) used chi-square tests. Abbreviation: Q, quintile.

* Includes those with a self-reported prior doctor-diagnosis of diabetes and screen-detected cases at baseline

† Incidence rate per 10,000 py was calculated from the adjusted HRs using a weighted method with the number of events in each group as the weighting variable

‡ Adjusted for age and sex-specific risk factors (top 5, as listed in the table)

## Data Availability

The CKB study is committed to sharing anonymised baseline, resurvey, and cause-specific mortality and morbidity data with bona fide researchers. All applications are reviewed by a Data Access Committee and data are shared unless data are being used for existing analyses. Details about data access policies and procedures are provided on the CKB website (www.ckbiobank.org).
