# Gender differences in modifiable risk factors for hip fracture: 10‐year follow‐up of a prospective study of 0.5 million Chinese adults

**DOI:** 10.1111/joim.13429

**Published:** 2021-12-22

**Authors:** Pang Yao, Sarah Parish, Derrick A. Bennett, Huaidong Du, Ling Yang, Yiping Chen, Yu Guo, Canqing Yu, Gang Zhou, Jun Lv, Liming Li, Zhengming Chen, Robert Clarke, China Kadoorie Biobank Collaborative Group

**Affiliations:** ^1^ Clinical Trial Service Unit and Epidemiological Studies Unit (CTSU) Nuffield Department of Population Health University of Oxford Oxford UK; ^2^ NIHR Oxford Biomedical Research Centre Oxford UK; ^3^ Medical Research Council Population Health Research Unit University of Oxford Oxford UK; ^4^ Fuwai Hospital Chinese Academy of Medical Sciences National Center for Cardiovascular Diseases Beijing China; ^5^ Department of Epidemiology School of Public Health Peking University Health Science Center Beijing China; ^6^ Peking University Center for Public Health and Epidemic Preparedness & Response Beijing China; ^7^ NCDs Prevention and Control Department Henan CDC Henan China

**Keywords:** CKB data release 15, hip fracture, incidence rate, population attributable fractions, risk factors

## Abstract

**Background:**

Little is known about the incidence rates and importance of major modifiable risk factors for hip and major osteoporotic fractures in low‐ and middle‐income countries. We estimated the age‐ and sex‐specific incidence of hip, major osteoporotic, and any fractures and their associated risk factors in Chinese adults.

**Methods:**

This was a prospective study of 512,715 adults, aged 30–79 years, recruited from 10 diverse areas in China from 2004 to 2008 and followed up for 10 years. Age‐ and sex‐specific incidence rates were estimated, and Cox regression was used to yield adjusted hazard ratios (HRs) and population attributable fractions for risk factors.

**Results:**

The incidence rates of hip fracture in Chinese adults were 5.1 (95% confidence interval [CI] 5.0–5.3) per 10,000 person‐years; they were higher in women than in men and increased by two‐ to threefold per 10‐year older age. Among men, five risk factors for hip fracture, including low education (HR = 1.23; 95% CI 1.04–1.45), regular smoker (1.22, 1.03–1.45), lower weight (1.59, 1.34–1.88), alcohol drinker (1.18, 1.02–1.36), and prior fracture (1.62, 1.33–1.98), accounted for 44.3% of hip fractures. Among women, lower weight (1.30, 1.15–1.46), low physical activity (1.22, 1.10–1.35), diabetes (1.62, 1.41–1.86), prior fracture (1.54, 1.33–1.77), and self‐rated poor health (1.29, 1.13–1.47) accounted for 24.9% of hip fractures. Associations of risk factors with major osteoporotic or any fractures were weaker than those with hip fractures.

**Conclusions:**

The age‐ and sex‐specific incidence rates of hip fracture in Chinese adults were comparable with those in Western populations. Five potentially modifiable factors accounted for half of the hip fractures in men and one quarter in women.

## Graphical abstract

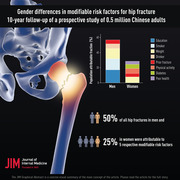

## Introduction

Osteoporosis is characterized by low bone mass and micro‐architectural deterioration of bone tissue, resulting in bone fragility and susceptibility to fracture [1]. Approximately one in two women and one in five men aged 50 years or older will experience an osteoporotic fracture in their remaining lifetime [2, 3, 4]. Hip fractures are the most serious type of osteoporotic fracture, with an approximate 30% absolute risk of death in the year following hip fracture [5]. Fractures of vertebrae, humerus, and forearm or hip are referred to as “major osteoporotic fractures” [6]. Both hip and major osteoporotic fractures are more common in women than in men and the incidence of both increases exponentially with age [7, 8].

Previous studies reported lower incidence rates of hip fractures in Asian populations than in Western populations, with reported age‐standardized incidence rates per 10,000 person‐years (py) of 14 in China, 13 in India, 44 in Denmark, 42 in Norway, 40 in Sweden, and 25 in the UK [9, 10]. The global burden of hip fractures is increasing worldwide, and current estimates suggest that the annual incidence will reach 4.5 million cases per year by 2050. About half of these cases are likely to occur in Asia, particularly in China [11, 12]. Previous city‐level studies of hospitalized cases have reported differences in the age‐ and sex‐specific incidence rates of hip fractures between different areas of China, albeit the reasons for such differences are uncertain [10, 13–16].

Several risk factors have been consistently associated with higher risk of hip fractures, including age, sex, weight, height, and prior history of fracture, but the relevance of diabetes, alcohol use, or socioeconomic status is uncertain [17, 18]. While gender differences in the incidence and mortality of hip fracture have been consistently reported [19], many previous studies of hip fracture have focused exclusively on women [17, 20, 21, 22, 23, 24] or on a single set of risk factors [21, 24, 25], and little is known about the relative importance of major modifiable risk factors in low‐ and middle‐income countries (LMICs), such as China.

The present report examines the incidence of hip and major osteoporotic fractures and their associated risk factors in a 10‐year follow‐up of a cohort study of >0.5 million adults recruited from 10 diverse areas in China [26]. The aims of this report were to (i) estimate the age‐ and sex‐specific incidence rates of fracture types (i.e., hip fracture, major osteoporotic fracture, and any fracture) overall and by areas within China, (ii) identify major risk factors for fracture types, and (iii) assess the population‐attributable fractions (PAF) for any potentially modifiable risk factors for hip fracture.

## Methods

### Study population

Details of the design and methods used for recruitment for the China Kadoorie Biobank (CKB) study have been previously reported [26]. Overall, 512,715 participants aged 30–79 years took part in the baseline survey between June 2004 and July 2008. Local, national, and international ethics approvals were obtained, and all participants provided written informed consent.

### Data collection

At the local study assessment clinics, participants completed an interviewer‐administered laptop‐based questionnaire that included questions on sociodemographic characteristics, smoking, alcohol consumption, diet, physical activity, personal and family medical history, and current use of medication. Physical measurements were recorded using calibrated instruments for height, weight, hip and waist circumference, bioimpedance, lung function, blood pressure, and heart rate (see Table S1 for details).

### Follow‐up for incident cases of fracture types

The vital status of each participant was determined periodically through China's Disease Surveillance Points (DSP) system and national health insurance systems, supplemented by annual active confirmation through street committees or village administrators [26]. Data on the incidence of major diseases and any hospitalizations were collected by linkage, using each participant's unique national identification number, with disease registries and national health insurance claims databases and all mortality registries. All deaths or hospital admissions were coded using the International Classification of Diseases, 10th Revision (ICD‐10) by trained DSP staff who were blinded to the other information collected in the study. By 1 January 2017, 44,066 (8.6%) participants died and 4751 (0.9%) were lost to follow‐up.

The primary outcomes were admission to hospital with hip fracture. The secondary outcomes included major osteoporotic fracture, any fracture, and osteoporosis (see the Appendix in Supporting Information for the ICD‐10 codes used to define disease endpoints) [27]. All analyses were restricted to known first‐ever hospitalization events for that outcome during the follow‐up period.

### Statistical analyses

All the analyses were performed separately for men and women. The incidence rates of fracture types were standardized by age and study area, with exposure time (years) calculated from the date of enrolment until the incident fracture, death, or censoring date (31 December 2016) for follow‐up. The incidence rates and their 95% confidence intervals (CIs) were estimated using the number of fracture cases per 10,000 py. The SEs were calculated assuming the number of cases with a Poisson distribution [28]. Cox proportional hazards models stratified by area were used to estimate the sex‐specific hazard ratios (HRs) for fracture types associated with individual risk factors in univariable and multivariable analyses (eAppendix). PAFs (expressed as a percentage) assuming a causal relationship were estimated for potentially modifiable risk factors (i.e., excluding age and height) separately in men and women [29, 30, 31]. Details of the methodology used to estimate PAFs are provided in Table S1 [32]. Collinearity between multiple risk factors was assessed using a variance inflation factor (VIF). A VIF factor >10 was used to indicate collinearity. In sensitivity analyses, we excluded individuals who reported a prior history of any fracture at baseline and fractures occurring during the first 5 years of follow‐up. All analyses were conducted using R version 3.6.2.

## Results

Among the 512,715 participants included, the mean age was 52 years and 59% were women. Compared with men, women were younger, less educated, had lower household income, and were much less likely to smoke (3.3% vs 74.2%) and drink alcohol (2.5% vs 37.0%). In contrast, women had a higher prevalence of overweight or obesity (45.3% vs 41.9%). The prevalence of a prior history of any fracture at enrolment was 8.8% in men and 5.7% in women (Table 1).

**Table 1:** Selected baseline characteristics in men and women

Characteristics	Overall(512,715)	Men(210,205)	Women (302,510)
Demographic factors			
Age, years	52.0 (10.7)	52.9 (10.9)	51.5 (10.5)
Urban residents, %	44.1	43.3	44.9
Education less than high school, %	79.0	73.9	82.5
Household income (>20,000 yuan/year), %	42.7	45.6	40.8
Lifestyle factors			
Regular smokers, %	32.4	74.2	3.3
Regular drinkers, %	16.7	37.0	2.5
Physical activity, MET h/day	21.1 (13.9)	22.4 (15.3)	20.2 (12.8)
Dietary factors			
Meat (≤3 days/week)	52.8	48.4	55.9
Fish (<1 day/week)	53.2	50.9	54.8
Fresh fruit (<1 day/week)	40.4	44.0	37.8
Dairy (nonconsumers)	68.4	69.3	67.8
Medical history and health status, %			
Poor self‐rated health	10.4	8.8	11.5
Diabetes^a^	5.9	5.5	6.3
History of fracture	6.9	8.8	5.7
History of rheumatoid arthritis	2.1	1.4	2.5
History of CVD^b^	4.5	4.8	4.3
History of cancer	0.5	0.4	0.5
Hypertension	33.5	35.8	31.9
Anthropometry			
Standing height, cm	158.9 (8.3)	165.3 (6.5)	154.1 (6.0)
Weight, kg	59.8 (10.8)	64.3 (10.9)	56.6 (9.5)
BMI, kg/m^2^	23.7 (3.4)	23.5 (3.2)	23.8 (3.5)
BMI ≥24 kg/m^2^, %	43.8	41.9	45.3
Waist—hip ratio	0.88 (0.07)	0.90 (0.06)	0.87 (0.07)
SBP, mmHg	131 (21)	132 (20)	130 (22)

*Note*: Mean (SD) and percentages were standardized by age at baseline (5‐year intervals) and area of the CKB population.

Abbreviations: BMI, body mass index; CHD, coronary heart disease; CKB, China Kadoorie Biobank; CVD, cardiovascular disease; MET‐h, metabolic equivalent of task‐hours; SBP, systolic blood pressure.

^a^ Includes those with a self‐reported diagnosis by a doctor of diabetes and screen‐detected cases at baseline.

^b^ Includes self‐reported diagnosis by a doctor of CHD, stroke, or transient ischemic attack.

During a median follow‐up of 10 years, a total of 15,762 participants were hospitalized for the first time with any fracture (2616 with hip fracture and 6857 with major osteoporotic fracture) and 2690 had a reported diagnosis of osteoporosis. The overall incidence rate of hip fracture per 10,000 py was 5.1 (95% CI 5.0–5.3) with higher rates in women than in men (5.8 [5.5–6.1] vs 4.2 [3.9–4.5]), and in rural than in urban areas (5.5 [5.2–5.8] vs 4.7 [4.5–5.0]) (Table 2). Across the 10 study areas, the age‐adjusted incidence rates of hip fracture varied by almost fivefold in men and 10‐fold in women (Fig. S1). Similar, albeit less extreme, associations were observed for major osteoporotic fracture, any fracture, and osteoporosis (Table 2).

**Table 2:** Standardized incidence rates (per 10,000 person‐years) of different types of fracture and osteoporosis

	Hip fracture		Major osteoporotic fracture**^a^**		Any fracture		Osteoporosis	
	No. ofevents	Rate (95% CI)	No. ofevents	Rate (95% CI)	No. ofevents	Rate (95% CI)	No. ofEvents	Rate (95% CI)
All	2616	5.1 (5.0–5.3)	6857	13.5 (13.2–13.9)	15,762	31.4 (30.9–31.9)	2690	5.3 (5.1–5.5)
Sex								
Men	863	4.2 (3.9–4.5)	1898	9.3 (8.9–9.7)	5472	27.0 (26.3–27.7)	515	2.5 (2.3–2.7)
Women	1753	5.8 (5.5–6.1)	4959	16.4 (16.0–16.9)	10,290	34.3 (33.7–35.0)	2175	7.2 (6.9–7.5)
Age at risk, years								
30–39	10	0.5 (0.2–0.8)	38	1.7 (1.2–2.4)	148	6.8 (5.8–8.0)	9	0.4 (0.2–0.8)
40–49	132	1.0 (0.8–1.1)	534	3.9 (3.6–4.3)	2170	16.0 (15.4–16.7)	161	1.2 (1.0–1.4)
50–59	331	2.1 (1.9–2.3)	1366	8.6 (8.1–9.0)	4339	27.4 (26.6–28.2)	497	3.1 (2.8–3.4)
60–69	664	5.5 (5.1–5.9)	2184	18.0 (17.3–18.8)	4881	40.8 (39.7–42.0)	892	7.3 (6.9–7.8)
70–79	1112	17.9 (16.9–19.0)	2185	35.5 (34.0–37.0)	3476	57.0 (55.1–58.9)	957	15.4 (14.5–16.4)
80+	367	55.4 (49.9–61.4)	550	84.3 (77.4–91.6)	748	116.5 (108.3–125.2)	174	26.2 (22.4–30.4)
Area								
Rural	1413	5.5 (5.2–5.8)	4052	15.2 (14.7–15.6)	10,757	39.6 (38.9–40.4)	1860	7.0 (6.7–7.4)
Urban	1203	4.7 (4.5–5.0)	2805	11.4 (10.9–11.8)	5005	21.0 (20.4–21.6)	830	3.3 (3.0–3.5)

*Note*: Standardized by age (10‐year intervals), sex, and study area (10 areas) of CKB population (where appropriate).

Abbreviations: CI, confidence interval; CKB, China Kadoorie Biobank.

^a^ Includes fractures of hip, vertebra, humerus, and ulna/radius.

**Fig. 1:**
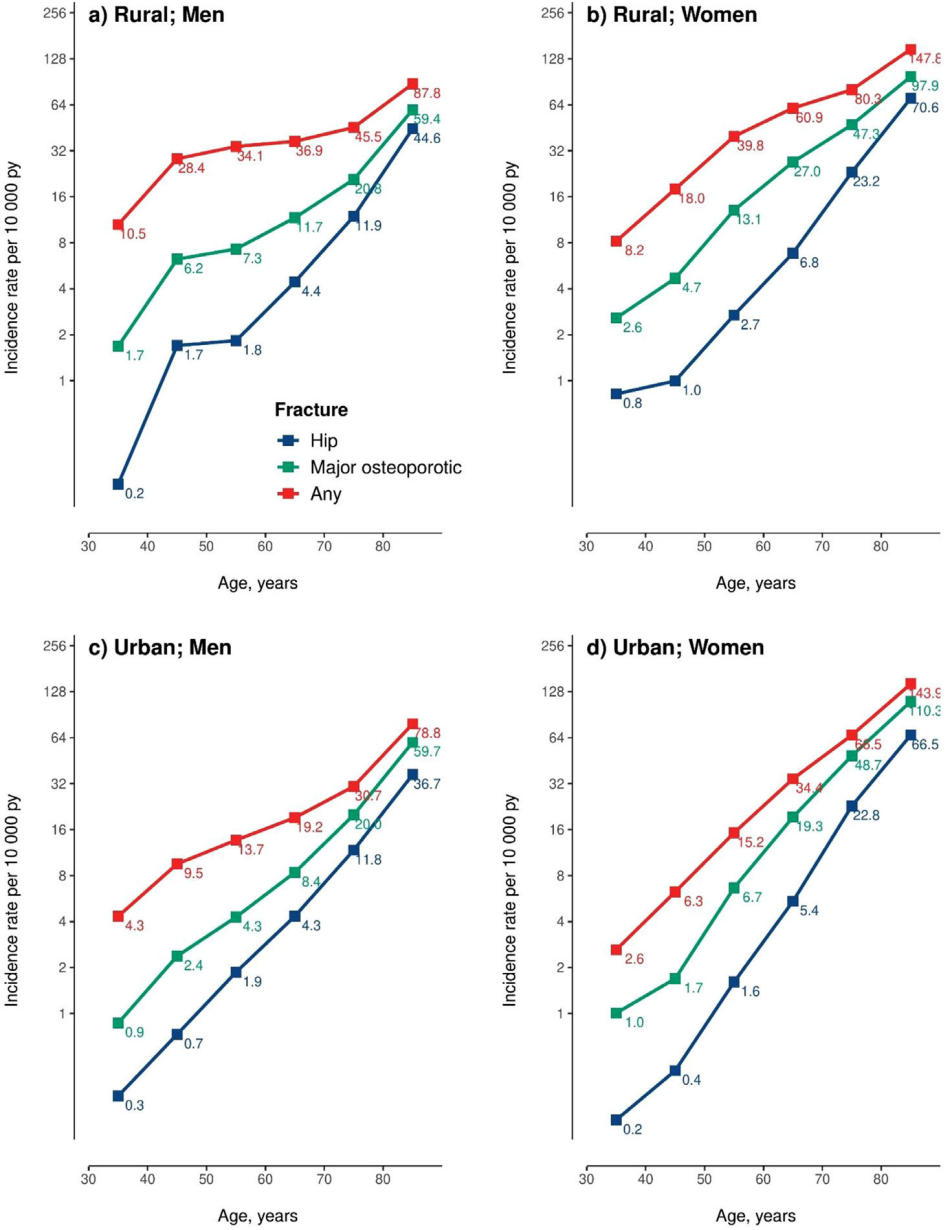
Age‐ and sex‐specific incidence rates of different fracture types, by area. The age‐specific incidence rates of different fracture types were estimated separately in (a) rural men, (b) rural women, (c) urban men, and (d) urban women. The numbers next to the squares are the incidence rates per 10,000 person‐years (pys).

The incidence rates of hip fracture were slightly higher in men than in women up until age 50 years, after which incidence rates increased much more rapidly in women than in men, increasing from 1.6 at 50–59 years to 66.5 per 10,000 py at 80 years or older in urban women and from 2.7 to 70.6 per 10,000 py in rural women (Fig. 1). Among men, the corresponding age‐related changes were much less extreme in both urban (varied from 1.9 to 36.7) and rural areas (from 1.8 to 44.6) (Fig. 1). Similarly, the proportion of hip fracture to any fracture increased rapidly with age, from 2%–9% at age 30–39 years to 46%–51% at age 80 years or older.

**Fig. 2:**
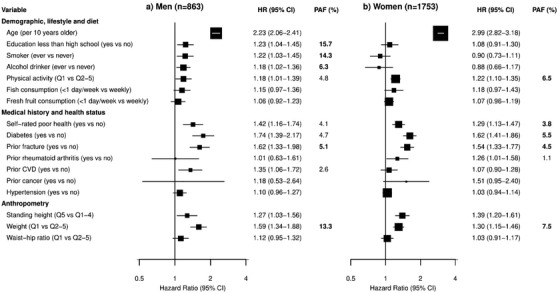
Associations of risk factors with hip fracture, by sex. Multivariable analyses after stratifying by area were used to explore the potential risk factors separately in men and women. The size of the squares is proportional to the inverse of the variance of the log hazard ratios (HRs). Population attributable fractions for hip fracture are presented for the modifiable risk factors. The population‐attributable fraction (PAF) values highlighted in bold are the top five risk factors in men and women. Abbreviation: Q, quintile.

Several major risk factors (e.g., low education, low physical activity, low consumption of fish or fresh fruit, and history of fracture) were also independently associated with the risk of hip fracture in univariable analyses in men and women (Table S2). In multivariable analyses (Fig. 2), age was more strongly associated with higher risk of hip fracture in women than in men (per 10‐year older: 2.99 [2.82–3.18] vs 2.23 [2.06–2.41]; *χ^2^* = 30.3, *P* = 2.2e‐08). Low physical activity, self‐rated poor health, diabetes, and history of fracture were each strongly associated with higher risks of hip fracture in both men and women (HR range: 1.18–1.74). Regular smoking or regular alcohol drinking were each associated with higher risks of hip fracture in men but not in women. Lower levels of education and prior Cardiovascular Disease were also associated with higher risks of hip fracture, but only in men. Prior rheumatoid arthritis was associated with a higher risk of hip fracture only in women.

Taller standing height was positively associated with a higher risk of hip fracture in both men and women (Fig. S2). The association of height with hip fracture was log‐linear, with each 1 standard deviation (SD) taller height associated with an adjusted HR of 1.07 (0.99–1.16) in men and 1.16 (1.10–1.23) in women. In contrast, all measures of adiposity, including weight, hip circumference, waist circumference, waist–hip ratio, waist–height ratio, and body mass index were each inversely associated with the risk of hip fracture (Fig. S2). Likewise, physical activity was inversely associated with hip fracture in both men and women (Fig. S3). Height, weight, and waist–hip ratio were selected for multivariable analyses (as these were less correlated with each other, with correlation coefficients <0.6), with the risk group being the top quintile for height, and bottom quintile for weight, waist–hip ratio, and physical activity. In multivariable analyses, taller standing height and lower weight were each strongly associated with higher risks of hip fracture in both men and women (Fig. 2). The associations of these risk factors with major osteoporotic fracture and any fracture were similar, albeit less extreme, as those with hip fracture (Fig. S4). Likewise, age, self‐rated poor health, history of fracture, or rheumatoid arthritis were also associated with higher risk of osteoporosis in men and women (Fig. S4). There was no evidence of collinearity between any of these risk factors and risk of hip fracture.

Figure 2 shows that the modifiable risk factors that accounted for the highest proportions of the PAF for hip fracture differed between men and women. Low education was the most important risk factor for hip fracture in men, accounting for 15.7% of the PAF, followed by regular smoker (14.3%), lower weight (13.3%), alcohol drinker (6.3%), prior fracture (5.1%), low physical activity (4.8%), diabetes (4.7%), self‐rated poor health (4.1%), and prior cardiovascular disease (CVD) (2.6%). Together, the top five modifiable factors based on the PAF accounted for 44.3% of all hip fractures in men. In women, lower weight accounted for 7.5% of hip fractures, followed by low physical activity (6.5%), diabetes (5.5%), prior fracture (4.5%), self‐rated poor health (3.8%), and prior rheumatoid arthritis (1.1%). Together, the top five modifiable factors accounted for 24.9% of all hip fractures in women.

Table 3 shows the distribution of the top five modifiable risk factors by 10 areas separately for men and women. Compared to the regional differences in the age‐adjusted incidence rates of hip fracture (∼fivefold in men and ∼10‐fold in women), the difference decreased to ∼fourfold in both men and women when further adjusted for the leading five risk factors. The associations of these risk factors with hip fracture were largely unaltered by the exclusion of participants with a prior history of fracture at baseline (Fig. S5), or by the exclusion of fractures occurring during the first 5 years of follow‐up (Fig. S6), or both (Fig. S7).

**Table 3:** Regional differences in incidence rates of hip fracture and their associated risk factors (top five) in men and women

Risk factors	Zhejiang	Liuzhou	Hunan	Sichuan	Suzhou	Gansu	Harbin	Haikou	Qingdao	Henan
Men										
No. of participants	24,027	19,321	26,370	21,315	22,363	19,298	23,252	10,794	15,624	27,841
Age‐adjusted rate^b^	6.3	5.3	3.8	3.8	3.6	3.3	2.9	2.5	2.1	1.3
Education less than high school	94.2	55.3	88.4	89.4	86.5	83.6	40.1	49.9	58.8	78.5
Ever‐regular smokers, %	82.3	65.3	78.1	78.5	81.6	78.4	68.6	52.6	74.0	71.9
Weight Q1, %	24.1	14.9	37.5	35.4	14.9	27.9	6.0	19.6	3.0	13.4
Ever‐regular drinkers, %	43.1	29.9	31.6	56.9	45.6	9.2	51.8	18.2	50.4	26.5
Prior fracture, %	11.3	12.1	8.1	5.4	18.4	3.9	6.9	4.0	3.8	9.6
Multivariable‐adjusted rate^c^	5.7	6.0	3.5	3.4	3.4	3.4	3.6	3.0	2.4	1.5
Women										
No. of participants	33,677	30,852	33,530	34,371	30,896	30,589	34,304	18,892	19,884	35,515
Age‐adjusted rate^b^	9.6	6.1	5.4	4.6	4.9	3.7	3.6	3.1	3.4	1.0
Weight Q1, %	23.3	18.0	34.0	32.0	16.6	22.5	9.1	25.4	3.1	12.1
Physical activity Q1, %	9.7	24.9	24.1	9.3	15.8	8.7	29.1	32.8	33.3	23.8
Diabetes, %^a^	5.8	8.3	4.1	4.0	5.3	3.6	9.6	6.6	10.4	5.7
Prior fracture, %	8.7	9.1	4.6	2.5	15.0	1.3	4.4	2.4	1.9	4.0
Self‐rated poor health, %	5.1	10.5	7.0	22.9	12.3	12.4	11.5	7.3	5.3	14.8
Multivariable‐adjusted rate^c^	7.8	8.1	4.7	4.6	4.7	4.6	4.8	4.1	3.3	2.0

*Note*: Areas are ordered by incidence rates of hip fractures in men. Comparisons of numbers (percentages) used chi‐square tests.

Abbreviation: py, person‐years; Q, quintile.

^a^ Includes those with a self‐reported prior doctor's diagnosis of diabetes and screen‐detected cases at baseline.

^b^ Incidence rate per 10,000 py was calculated from the adjusted hazard ratios using a weighted method with the number of events in each group as the weighting variable.

^c^ Adjusted for age and sex‐specific risk factors (top five, as listed in the table).

## Discussion

This study demonstrated that the incidence rates of hip fracture in Chinese adults were comparable with those in European populations [5], and were higher in women than in men at age 50 years or older. The incidence rates for hip fracture at age ≥80 years were 40.3 in men and 68.2 per 10,000 py in women, while in the UK, the corresponding rates obtained from primary care records were 40.1 and 89.4, respectively [7]. Overall, five potentially modifiable risk factors accounted for about half of all hip fractures in men and a quarter in women. Several modifiable markers of frailty (low weight and low physical activity) and adverse lifestyle factors (smoking, alcohol use, and, particularly in men, low education) and medical history (diabetes, prior fracture, or rheumatoid arthritis) accounted for most hip fractures.

The incidence of hip fracture varied by five‐ to 10‐fold between different areas in China, which largely reflected differences between levels of potentially modifiable risk factors across these areas in men and women. However, it was not possible to fully exclude the possibility that differences in health systems may account for some of these geographic differences. The China Health and Retirement Longitudinal Study also reported that the incidence of hip fracture was higher in Zhejiang, Sichuan, and Guangxi provinces than in other areas in China [33].

Previous studies on hip fracture were limited to city‐level studies of hospitalized cases and were also constrained by a small sample size, short duration of follow‐up, and limited coverage of geographic areas within China [10, 13—16]. One prospective study reported higher incidence rates of hip fracture among urban adults (age ≥55 years, 9.9–12.2 and 15.6–20.4 per 10,000 py in men and women, respectively) than the CKB study (5.5 and 8.2 per 10,000 py) [34], but the mean age of the latter study participants was considerably older than in the present study (77.1 vs 60.0 years). A systematic review of the worldwide incidence of hip fracture reported lower incidence rates of hip fracture in Chinese than in Western populations [9]. Several studies have reported stabilized or declining incidence rates of hip fracture in North America [8, 28, 35], Europe [36], and in Hong Kong and Singapore (two economically advanced cities in Asia) [37], but rates of hip fracture in LMICs, such as China, appear to be increasing [10, 38]. Both the increasing incidence rates of fracture and higher proportions of the population that survive to old age highlight the need for more effective strategies for the prevention of fractures.

In contrast with previous studies in China, which mainly used cross‐sectional or case‐control study designs [18, 25, 33] or were restricted to Chinese postmenopausal women [21], the present study provides a detailed evaluation of potentially modifiable risk factors for hip fracture in Chinese men and women independently. Consistent with current fracture risk assessment tools [39], increasing age, taller height, lower weight, and prior diabetes or fracture were independent risk factors for hip fractures in both men and women. The association of height with risk of hip fracture observed in the present study probably reflects biomechanical mechanisms [40]. The length of the femur is a determinant of the fracture risk after a fall, and individuals with taller height require less force to sustain a fracture. Individuals with higher levels of adiposity are believed to have greater physical protection from a higher mass of gluteofemoral adipose tissue, which reduces the impact of falling and subsequent risk of fracture [41]. Low weight, a marker of frailty, is a risk factor for hip fracture, and this effect may be mediated through low bone mineral density (BMD), as decreased muscle mass and strain may decrease BMD and the structural integrity of the underlying bones [41].

Low levels of education were associated with the highest PAF of the risk factors in men. An inverse association between the highest level of educational attainment and the risk of hip fracture was previously reported in some studies in Europe and the USA [42] and in China [33], but not in others [41]. It is possible that people with lower educational attainment may have more unhealthy lifestyles and are less likely to undergo screening for bone and other diseases, and suffer higher risks of falls.

Low physical activity has also been associated with higher risk of different fracture types in several previous studies, which may reflect the effects of frailty [24]. Higher levels of physical activity may reduce the risks of hip fracture by improving balance, coordination, and muscle strength, but participation in physical activity may also increase the risks of falls, injury, and other fractures [23, 24, 43]. Other studies [23, 24] have also reported that higher levels of physical activity were associated with lower risk of hip fracture, but higher risks of knee, elbow, ankle, or wrist fracture [23, 24].

Previous studies in Western populations have also demonstrated the importance of current status as cigarette smoker and alcohol drinker as independent risk factors for hip fractures in women [17, 44], in men [45, 46], or both [18]. However, in CKB, few Chinese women regularly smoked tobacco or drank alcohol. The strength of the associations for current smoking in men (HR 1.22) was similar to that in a previous study in Singapore (1.23 for men and 1.27 for women) [47], but lower than in previous studies in Western populations [45, 46]. Nevertheless, approximately 15% of all hip fractures in the present study population were attributable to tobacco smoking, consistent with previous estimates of 19% in Western studies [48].

The lack of association of hip fracture with rheumatoid arthritis (RA) may reflect the smaller number of men with RA. History of CVD was not associated with a higher risk of hip fracture in the multivariable analyses, perhaps reflecting confounding by low levels of physical activity and diabetes. Consistent with previous reports in Western populations, both men and women with a self‐reported prior history of poor health in the present study had a higher risk of hip fracture, possibly reflecting effects of frailty, due to underlying disease or treatment [49].

The present study had several strengths, including prospective study design, large numbers of well‐characterized participants enrolled from 10 diverse areas, and the ability to assess incident cases of different fracture types. The study also had several limitations, including not being representative of the Chinese population; however, this does not preclude the generalizability of the relative risks with individual risk factors [50]. The available evidence collected on osteoporosis was limited to those identified during admission to hospital, rather than any systematic screening for osteoporosis. Importantly, many of the observed risk factor associations for hip and major osteoporotic fractures were concordant with those reported by previous studies in European populations. Public health strategies for prevention of hip and major osteoporotic fracture should target older people with selected markers of frailty (low weight and low physical activity) and the presence of other potentially modifiable risk factors (smoking, alcohol use and, particularly in men, low education) to reduce the morbidity and mortality associated with hip and major osteoporotic fractures worldwide.

## Author contributions

Pang Yao, Zhengming Chen, and Robert Clarke had full access to the data in the study and take responsibility for the integrity of the data and the accuracy of the data analysis. Zhengming Chen and Robert Clarke contributed equally to this report. Acquisition, analysis, or interpretation of data: Pang Yao, Sarah Parish, Zhengming Chen, and Robert Clarke. Pang Yao wrote the first draft of the manuscript. Critical revision of the manuscript for important intellectual content: all authors. Statistical analysis: Pang Yao. Administrative, technical, or material support: Derrick A. Bennett, Huaidong Du, Ling Yang, Yiping Chen, Yu Guo, Canqing Yu, Jun Lv, Liming Li, Gang Zhou, and Zhengming Chen. Supervision: Zhengming Chen and Robert Clarke.

## Conflict of interests

None of the authors had any conflict of interest in relation to this report.

## Supporting information

Supporting information

## Data Availability

The CKB study is committed to sharing anonymized baseline, resurvey, and cause‐specific mortality and morbidity data with bona fide researchers. All applications are reviewed by a Data Access Committee and data are shared unless data are being used for existing analyses. Details about data access policies and procedures are provided on the CKB website (www.ckbiobank.org).
