# Supplementary material for: Gender differences in modifiable risk factors for hip fracture: 10-year follow-up of a prospective study of 0.5 million Chinese adults
Source: J Intern Med. Author manuscript; Available in PMC 2022 Apr 1. (PMC7612528; doi:10.1111/joim.13429)

Supplementary Material

Gender differences in modifiable risk factors for hip fracture: 10-year follow-up of a prospective study of 0.5 million Chinese adults

Contents

eAppendix 2

Members of the China Kadoorie Biobank Collaborative Group 5

Sources of Funding 6

eTable 1. Classification of risk factors for estimation of PAF associated with hip fracture 7

eTable 2. Univariable analyses of risk factors for hip fracture 8

eFigure 1. Age-adjusted incidence rates of (a) fractures and (c) osteoporosis across 10 areas (b), by sex 9

eFigure 2. Association of anthropometry traits with hip fracture in men and women 10

eFigure 3. Association of physical activity with hip fracture in men and women 11

eFigure 4. Associations of risk factors with different types of fracture and osteoporosis in men and women 12

eFigure 5. Associations of risk factors with hip fracture in men and women, after excluding participants with history of fracture 14

eFigure 6. Associations of risk factors with hip fracture in men and women, after excluding the first 5 years of follow-up 15

eFigure 7. Associations of risk factors with hip fracture in men and women, after excluding participants with history of fracture, and the first 5 years of follow-up 16

eFigure 8. Age- and sex-specific incidence rates of fractures in CKB and UK 17

eAppendix

Study population

The study design was a prospective study. Participants who were aged 30 -79 years at enrolment, were selected for the China Kadoorie Biobank study of the causes of major chronic diseases that account for most deaths in the Chinese population. Overall, 515 681 people attended the baseline survey between June 2004 and July 2008, of whom 261 (0.05%) withdrew before completion, 2208 (0.4%) were found subsequently to have inadvertently attended the survey twice at different time points and 1 had major data errors. The estimated population response rate was ∼30% (26–38% in the five rural areas and 16–50% in the five urban areas). All individuals who completed a questionnaire and provided consent for participants in the study were included in the present analyses. No participants with a prior history of cardiovascular disease or cancer were excluded. While the China Kadoorie Biobank is not representative of the Chinese population, it collected data from a large number of participants from 5 rural and 5 urban areas that reflected a wide range of exposures and disease outcomes. The study participants did not receive any payment for their participation in the study.

Exposures

The study procedures, involving electronic data collection, ensured that there was virtually no missing data on covariates. While data were collected on the number of pregnancies, age at menarche and age at menopause, but not on use of hormone replacement therapy, these data were not included in the present analyses which focussed on common risk factors available in both men and women. Information was also collected on daily intake of fruit, vegetables, meat, fish and soy< but were not included in the present analyses. Covariates did include rheumatoid arthritis, but not osteoarthritic or non-specific arthritis as these were unrelated to risk of fracture. In the absence of any screening for osteoporosis (using bone imaging) in the study population, it was not possible to use osteoporosis as a risk factor (exposure) in this population. No data were available on the sites of fractures prior to enrolment.

Outcomes

Data on incidence of fracture and osteoporosis involved all hospital admissions were obtained by linkage to national health insurance claims databases and coded using the International Classification of Diseases, 10th Revision (ICD-10). There were no procedures in place for screening for osteoporosis in this study population. No data were collected on use of medication such as treatments for osteoporosis or chemotherapy for cancer that might alter risks of fracture. The study methodology and procedures for reporting of fracture outcomes were standardized for all 10 study areas. Follow-up for fracture incidence and all other disease outcomes were remarkably complete with <1% missing data. No data were available on the accuracy of reported fractures, but the reporting and adjudication accuracy of total stroke or ischaemic heart disease was over 95% in this study population.

The primary outcomes were admission to hospital with hip fracture (ICD-10 codes: S72.0-S72.2, S72.9).[1] The secondary outcomes included admission to hospital with a major osteoporotic fracture (hip, vertebra [S12.0-S12.7, S12.9, S22.0-S22.1, S32.0, S32.2, S32.7, S32.8], humerus [S42.2-S42.4, S42.7], radius/ulna [S52.0-S52.9]) or any fracture (S02-S92, M80). In addition, admissions to hospital with osteoporosis that were reported by health insurance coding system (M80–M82) were analysed independently of fracture incidence.

Statistical analyses

Cox Proportional Hazards models were used to estimate the sex-specific hazard ratios (HR) for fracture types associated with individual risk factors, before and after adjustment for other covariates. In univariable analyses, models were stratified by area and adjusted for age. All variables that were significantly associated ($P<0.05$) with hip fracture in the univariable analyses were subsequently assessed in multivariable analyses after stratifying by area. Cox proportional hazards models were used to assess the shape and strength of the associations between continuous traits (sex-specific quintiles of height, weight, hip and waist circumference, waist-hip ratio, waist-height ratio, BMI and physical activity) and risk of hip fracture risk. For Cox Proportional hazards, the assumption of proportionality was tested and met.

Population attributable fractions (PAFs, expressed as a percentage) assuming a causal relationship were estimated for potentially modifiable risk factors (i.e.,

excluding age and height), separately in men and women. PAF was calculated using a standard formula: PAF = P_d×(R−1)/R, where P_d was the proportion of fracture cases exposed to the risk factor, and R was the estimated HR associated with each risk factor in the present study.[2-4] Additional details about the methodology used to estimate PAFs are provided in **eTable 1.**

The incidence rates of hip fracture by areas (n=10) were also adjusted for age and levels of five leading (i.e., highest PAF) risk factors associated with hip fracture, using a previously reported formula: $IR_i = HR_i \times \frac{overall\ IR}{\frac{\sum W_i \times HR_i}{\sum W_i}}$, where IR was the incidence rate and W_i was the number of events in each area.[5]

Reference

1. Peng K, Yao P, Yang L, et al. Parenthood and risk of hip fracture: a 10-year follow-up prospective study of middle-aged women and men in China. *Osteoporos Int.* 2019.
2. Yusuf S, Joseph P, Rangarajan S, et al. Modifiable risk factors, cardiovascular disease, and mortality in 155 722 individuals from 21 high-income, middle-income, and low-income countries (PURE): a prospective cohort study. *The Lancet.* 2019.
3. Flegal KM, Panagiotou OA, Graubard BI. Estimating population attributable fractions to quantify the health burden of obesity. *Ann Epidemiol.* 2015;25(3):201-207.
4. Rockhill B, Newman B, Weinberg C. Use and misuse of population attributable fractions. *Am J Public Health.* 1998;88(1):15-19.
5. Liu X, Bragg F, Yang L, et al. Smoking and smoking cessation in relation to risk of diabetes in Chinese men and women: a 9-year prospective study of 0.5 million people. *Lancet Public Health.* 2018;3(4):E167-E176.

Members of the China Kadoorie Biobank Collaborative Group

International Steering Committee: Junshi Chen, Zhengming Chen (PI), Robert Clarke, Rory Collins, Yu Guo, Liming Li (PI), Jun Lv, Richard Peto, and Robin Walters.

International Co-ordinating Centre, Oxford: Daniel Avery, Derrick Bennett, Ruth Boxall, Fiona Bragg, Sushila Burgess, Kahung Chan, Yumei Chang, Yiping Chen, Zhengming Chen, Robert Clarke, Huaidong Du, Zammy Fairhurst-Hunter, Wei Gan, Simon Gilbert, Alex Hacker, Parisa Hariri, Michael Holmes, Andri Iona, Becky Im, Maria Kakkoura, Christiana Kartsonaki, Rene Kerosi, Garry Lancaster, Kuang Lin, John McDonnell, Iona Millwood, Qunhua Nie, Alfred Pozaricki, Paul Ryder, Sam Sansome, Dan Schmidt, Rajani Sohoni, Rebecca Stevens, Iain Turnbull, Robin Walters, Lin Wang, Neil Wright, Ling Yang, Xiaoming Yang and Pang Yao.

National Co-ordinating Centre, Beijing: Zheng Bian, Yu Guo, Xiao Han, Can Hou, Chao Liu, Jun Lv, Pei Pei, Canqing Yu and Chun Li.

Regional Co-ordinating Centres: **Qingdao** CDC: Zengchang Pang, Ruqin Gao, Shanpeng Li, Shaojie Wang, Yongmei Liu, Ranran Du, Liang Cheng, Xiaocao Tian, Hua Zhang, Yaoming Zhai, Feng Ning, Xiaohui Sun, Feifei Li. Licang CDC: Silu Lv, Junzheng Wang, Wei Hou. **Heilongjiang** Provincial CDC: Mingyuan Zou, Shichun Yan, Xue Zhou. Nangang CDC: Bo Yu, Yanjie Li, Qinai Xu, Quan Kang, Ziyan Guo. **Hainan** Provincial CDC: Ximin Hu, Jinyan Chen, Xiaohuan Wang. Meilan CDC: Min Weng, Zhendong Guo, Shukuan Wu, Yilei Li, Huimei Li. **Jiangsu** Provincial CDC: Ming Wu, Yonglin Zhou, Jinyi Zhou, Ran Tao, Jie Yang, Jian Su. Suzhou CDC: Fang Liu, Jun Zhang, Yihe Hu, Yan Lu, Liangcai Ma, Aiyu Tang, Yujie Hua, Jianrong Jin, Jingchao Liu. **Guangxi** Provincial CDC: Zhenzhu Tang, Naying Chen, Duo Liu. Liuzhou CDC: Mingqiang Li, Jinhuai Meng, Rong Pan, Qilian Jiang, Jian Lan, Yun Liu, Liuping Wei, Liyuan Zhou, Ningyu Chen, Ping Wang, Fanwen Meng, Yulu Qin, Sisi Wang. **Sichuan** Provincial CDC: Xianping Wu, Ningmei Zhang, Xiaofang Chen, Xunfu Zhong, Jiaqiu Liu, Qiang Sun. Pengzhou CDC: Guojin Luo, Jianguo Li, Xiaofang Chen, Xunfu Zhong, Jiaqiu Liu, Qiang Sun. **Gansu** Provincial CDC: Pengfei Ge, Xiaolan Ren, Caixia Dong. Maiji CDC: Hui Zhang, Enke Mao, Zhongxiao Li, Tao Wang, Xi Zhang. **Henan** Provincial CDC: Ding Zhang, Gang Zhou, Shixian Feng, Liang Chang, Lei Fan. Huixian CDC: Yulian Gao, Tianyou He, Huarong Sun, Pan He, Chen Hu, Xukui Zhang. **Zhejiang** Provincial CDC: Min Yu, Ruying Hu, Hao Wang, Weiwei Gong, Meng Wang. Tongxiang CDC: Chunmei Wang, Xiaoyi Zhang, Kaixu Xie, Lingli Chen, Dongxia Pan, Qijun Gu. **Hunan** Provincial CDC: Yuelong Huang, Biyun Chen, Li Yin, Huilin Liu, Zhongxi Fu, Qiaohua Xu. Liuyang CDC: Xin Xu, Hao Zhang, Huajun Long, and Libo Zhang.

Sources of Funding

The chief acknowledgment is to the participants, the project staff, staff of the China CDC and its regional offices for access to death and disease registries. The Chinese National Health Insurance scheme provided electronic linkage to all hospitalization data. The China Kadoorie Biobank study is jointly coordinated by the University of Oxford and the Chinese Academy of Medical Sciences. The funding body for the baseline survey was the Kadoorie Charitable Foundation, Hong Kong, China and the funding sources for the long-term continuation of the study include UK Wellcome Trust (212946/Z/18/Z, 202922/Z/16/Z, 104085/Z/14/Z, 088158/Z/09/Z), National Natural Science Foundation of China (81390540, 81390541, 81390544), and National Key Research and Development Program of China (2016YFC 0900500, 0900501, 0900504, 1303904). Core funding was provided to the CTSU, University of Oxford, by the British Heart Foundation, the UK Medical Research Council, and Cancer Research UK.

eTable 1. Classification of risk factors for estimation of PAF associated with hip fracture

Variable	Definition or method of measurement	Risk group	Reference group	Reference group (%)
**Demographic, lifestyle and diet**				
Education	Education was self-reported, and classified as Low (middle school education level or less), or High (high school, technical school/college, or university education)	Low education	High education	21
Smoking	Self-reported tobacco consumption using a standard tobacco use frequency questionnaire, categorized as never, occasional, ex-regular and regular smoker	Ever regular (ex-regular and regular smoker)	Definition of ever-regular was not met	68
Alcohol	Self-reported alcohol consumption using a standard alcohol consumption frequency questionnaire. Consumption was categorized as never regular, ex-regular, occasional, monthly, reduced intake or weekly drinker	Ever regular (ex-regular and weekly drinker)	Definition of ever-regular was not met	83
Physical activity	Physical activity was measured using the International Physical Activity Questionnaire,and calculate total physical activity in MET hours per day (MET-h/d)	Lowest quintile*	Quintiles 2-5*	80
Meat	Respondents were asked about the frequency of habitual consumption during the previous 12 months and chose among five categories of frequency: daily (29.3%), 4-6 days/week (17.9%), 1-3 days/week (35.5%), monthly (12.5%), never/rarely (4.8%)	Low consumption (⩽3 days/week)	High consumption (≥4 days/week)	52.8
Fish	Respondents were asked about the frequency of habitual consumption during the previous 12 months and chose among five categories of frequency: daily (2.8%), 4-6 days/week (6.1%), 1-3 days/week (37.9%), monthly (21.2%), never/rarely (32.0%)	Low consumption (<1 day/week)	High consumption (weekly)	53.2
Fresh fruit	Respondents were asked about the frequency of habitual consumption during the previous 12 months and chose among five categories of frequency: daily (18.8%), 4-6 days/week (9.4%), 1-3 days/week (31.5%), monthly (34.0%), never/rarely (6.4%)	Low consumption (<1 day/week)	High consumption (weekly)	40.4
Dairy	Respondents were asked about the frequency of habitual consumption during the previous 12 months and chose among five categories of frequency: daily (9.8%), 4-6 days/week (2.1%), 1-3 days/week (8.6%), monthly (11.1%), never/rarely (68.4%)	Non-consumers	Consumers	68.4
**Medical history and health status**				
Self-rated poor health	Self-rated health status categorized as Excellent, Good, Fair or Poor	Poor health (Yes)	No (Excellent/Good/Fair)	90
Diabetes	Diabetes was defined as self-reported history of diabetes or screen detected diabetes. Screen-detected diabetes was defined as no self-reported diabetes with a blood glucose level ≥7.0 mmol/l and a fasting time >8 h, a blood glucose level ≥11.1 mmol/l and a fasting time <8 h, or a fasting blood glucose level ≥7.0 mmol/l.	Yes	No	94
Prior fracture	Self-reported history of fracture	Yes	No	93
Prior rheumatoid arthritis	Self-reported history of rheumatoid arthritis	Yes	No	98
Prior CVD (yes vs no)	Self-reported history of IHD, stroke or TIA	Yes	No	95
Prior cancer (yes vs no)	Self-reported history of cancer	Yes	No	99
Hypertension	Defined as SBP≥140 mm Hg, or DBP≥90 mm Hg, or receiving treatment for hypertension	Yes	No	66
**Anthropometry**				
Standing height	Standing height was measured to the nearest 0.1 cm by using a stadiometer	Highest quintile*	Quintile 1-4*	80
Weight	Weight was measured to the nearest 0.1 kg using a TANITA TBF-300GS body composition analyzer (Tanita Corp., Tokyo, Japan)	Lowest quintile*	Quintiles 2-5*	80
Waist-hip ratio	Waist circumference was measured with a soft non-stretchable tape midway between the lowest rib and the iliac crest, also to the nearest 0.1 cm. The waist-hip ratio is calculated as waist measurement divided by hip measurement	Lowest quintile*	Quintiles 2-5*	80

* sex-specific quintiles

eTable 2. Univariable analyses of risk factors for hip fracture

	Men		Women	
**Variable**	**HR (95% CI)**	***P* value**	**HR (95% CI)**	***P* value**
**Demographic, lifestyle and diet**				
Age at risk (per 10 years increase)	2.43 (2.27-2.61)	<0.001	3.23 (3.07-3.40)	<0.001
Education less than high school (yes vs no)	1.32 (1.08-1.62)	0.007	1.08 (0.91-1.29)	0.380
Household income (CNY, ≥20k vs <20k)	0.96 (0.82-1.12)	0.590	1.05 (0.94-1.16)	0.400
Smoker (ever vs never)	1.34 (1.13-1.58)	<0.001	0.92 (0.75-1.13)	0.430
Alcohol drinker (ever vs never)	1.22 (1.06-1.40)	0.006	0.85 (0.64-1.13)	0.260
Physical activity (Quintile 1 vs Quintile 2-5)	1.22 (1.04-1.44)	0.014	1.26 (1.14-1.40)	<0.001
Meat (≤3 days/week vs ≥4 days/week)	1.10 (0.94, 1.27)	0.230	1.01 (0.91, 1.13)	0.810
Fish (<1 day/week vs weekly)	1.21 (1.02, 1.43)	0.026	1.21 (1.08, 1.36)	<0.001
Fresh fruit (<1 day/week vs weekly)	1.20 (1.03, 1.38)	0.016	1.15 (1.04, 1.28)	0.009
Dairy (non-consumers vs consumers)	1.01 (0.86, 1.20)	0.890	1.00 (0.89, 1.13)	0.970
**Medical history and health status**				
Self-rated poor health (yes vs no)	1.69 (1.39-2.05)	<0.001	1.45 (1.28-1.65)	<0.001
Diabetes (yes vs no)	1.73 (1.40-2.15)	<0.001	1.66 (1.45-1.90)	<0.001
Prior fracture (yes vs no)	1.64 (1.35-2.00)	<0.001	1.53 (1.32-1.76)	<0.001
Prior rheumatoid arthritis (yes vs no)	1.06 (0.66-1.69)	0.810	1.30 (1.04-1.63)	0.020
Prior CVD (yes vs no)	1.47 (1.17-1.86)	0.001	1.18 (0.99-1.40)	<0.001
Prior cancer (yes vs no)	1.34 (0.60-3.01)	0.470	1.59 (1.01-2.53)	0.044
Hypertension (yes vs no)	1.07 (0.93-1.23)	0.330	0.97 (0.92-1.00)	0.050
**Anthropometry**				
Standing height (Quintile 5 vs Quintile 1-4)	1.14 (0.94-1.40)	0.190	1.33 (1.15-1.54)	<0.001
Weight (Quintile 1 vs Quintile 2-5)	1.58 (1.36-1.84)	<0.001	1.23 (1.11-1.37)	<0.001
Waist-hip ratio (Quintile 1 vs Quintile 2-5)	1.26 (1.09-1.46)	0.002	1.08 (0.96-1.22)	0.183

Cox regression models stratified by area, and adjust for age (where available)
*Includes those with a (self-reported) previous medical diagnosis of diabetes and those detected by blood glucose tests at baseline

eFigure 1. Age-adjusted incidence rates of (a) fractures and (c) osteoporosis across 10 areas (b), by sex

Open circles indicate rural areas and solid circles indicate urban areas.

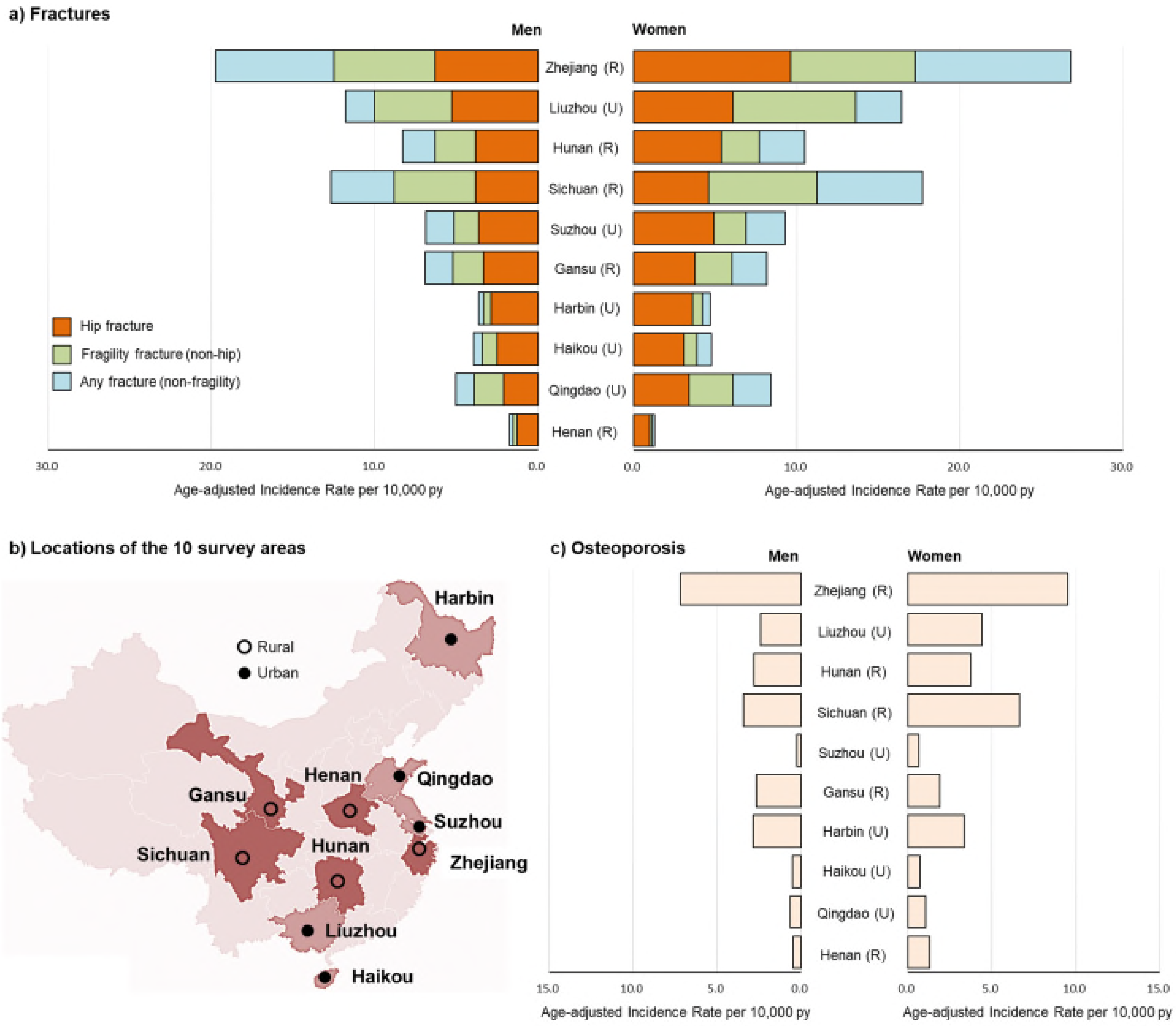

eFigure 2. Association of anthropometry traits with hip fracture in men and women

Risk factor levels for hip fracture were classified by quintiles in men (top panel) and women (bottom panel) separately. Hazard ratios (HRs) were plotted against the mean level in each group. The sizes of the data markers are proportional to the inverse of the variance of the log HRs. The numbers above the 95% CI are point estimates for HRs, and the numbers below are numbers of fracture cases for each category. The models were stratified by age at risk and region, and adjusted for age at baseline, education, household income, smoking and alcohol.

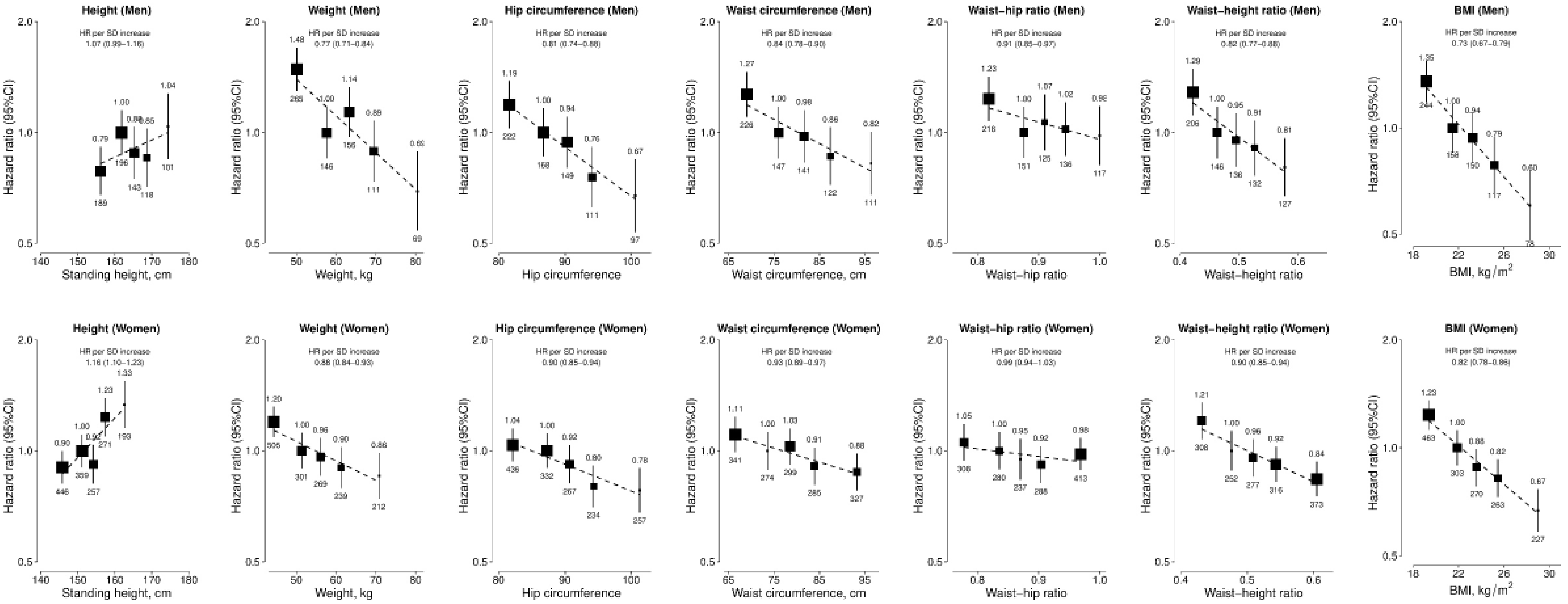

eFigure 3. Association of physical activity with hip fracture in men and women

Risk factor levels for hip fracture were classified by quintiles in men and women, separately. Hazard ratios (HRs) were plotted against the mean level in each group. The sizes of the data markers are proportional to the inverse of the variance of the log HRs. The numbers above the 95% CI are point estimates for HRs, and the numbers below are numbers of fracture cases for each category. The models were stratified by age at risk and region, and adjusted for age at baseline, education, household income, smoking and alcohol.

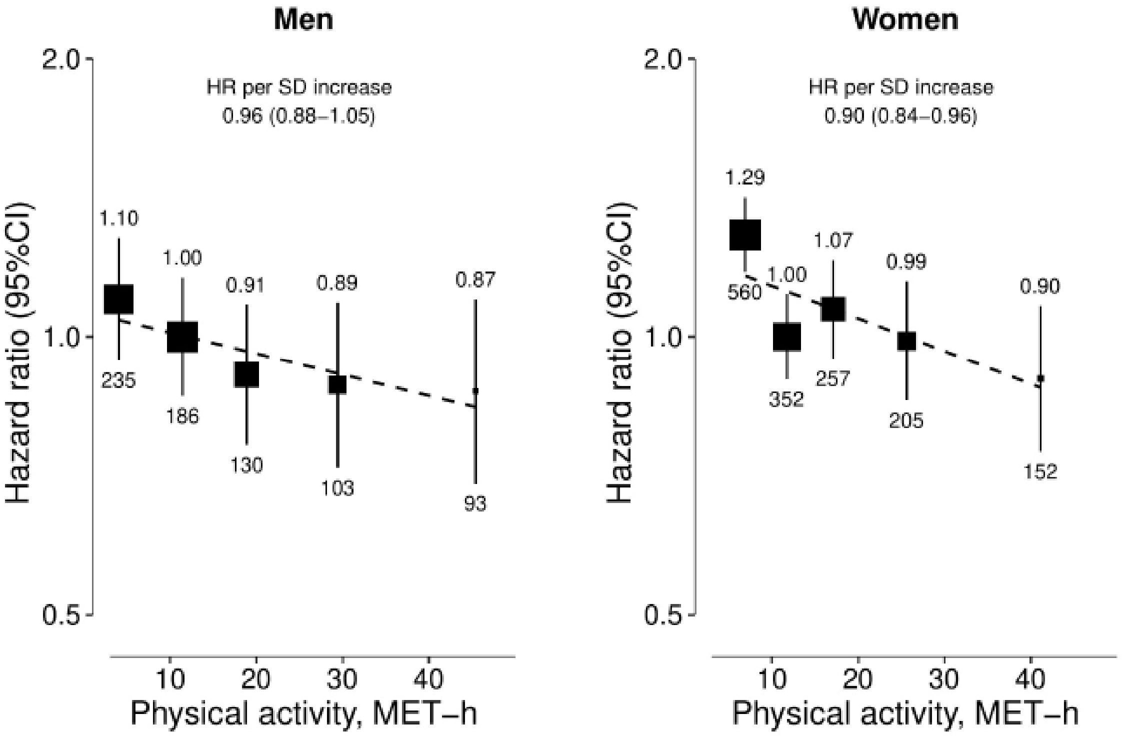

eFigure 4. Associations of risk factors with different types of fracture and osteoporosis in men and women

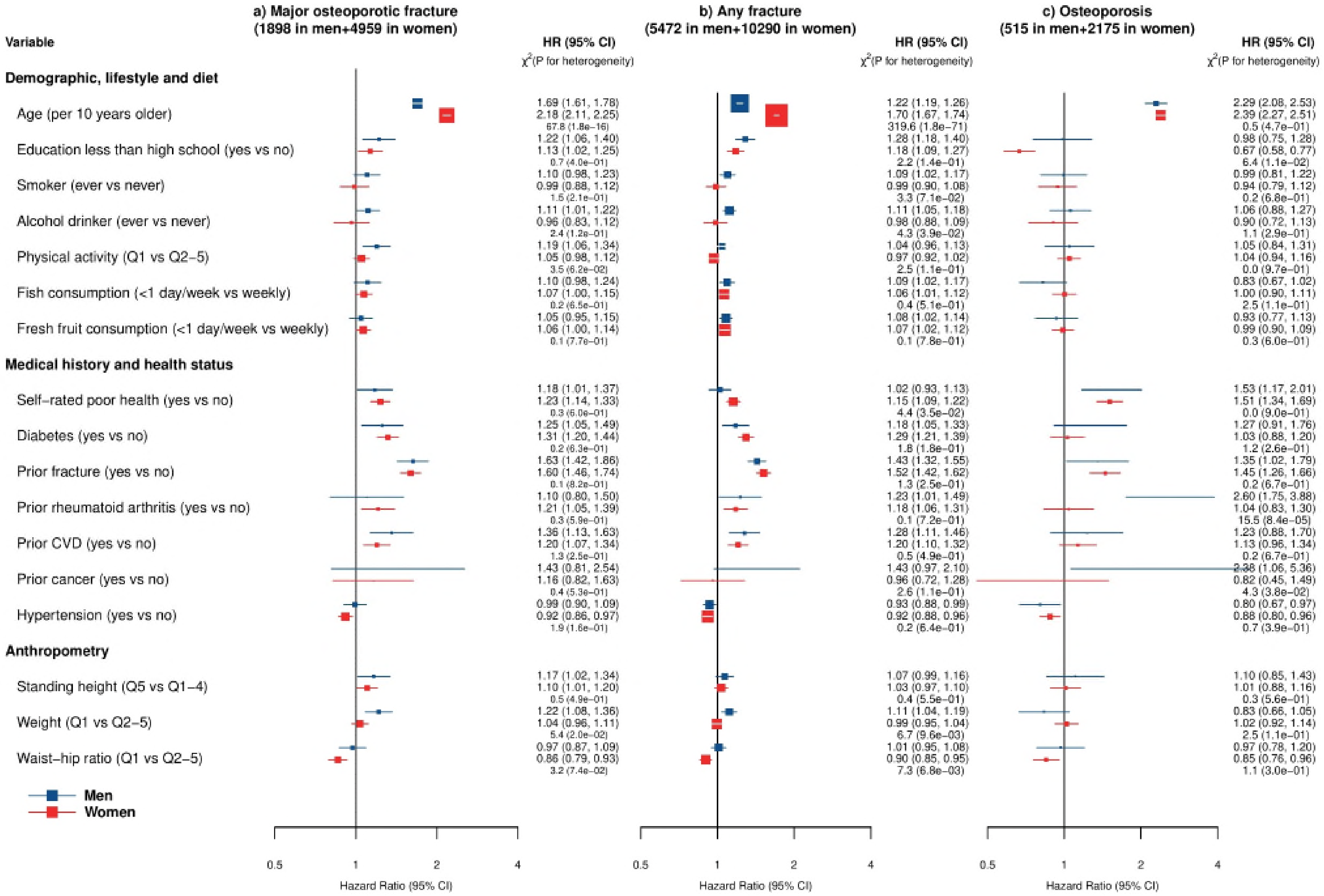

Variable
a) Major osteoporotic fracture
(1898 in men+4959 in women)
HR (95% CI)
χ²(P for heterogeneity)
b) Any fracture
(5472 in men+10290 in women)
HR (95% CI)
χ²(P for heterogeneity)
c) Osteoporosis
(515 in men+2175 in women)
HR (95% CI)
χ²(P for heterogeneity)
Demographic, lifestyle and diet
Age (per 10 years older)
1.69 (1.61, 1.78)
2.18 (2.11, 2.25)
67.8 (1.8e−16)
1.22 (1.19, 1.26)
1.70 (1.67, 1.74)
319.6 (1.8e−71)
2.29 (2.08, 2.53)
2.39 (2.27, 2.51)
0.5 (4.7e−01)
Education less than high school (yes vs no)
1.22 (1.06, 1.40)
1.13 (1.02, 1.25)
0.7 (4.0e−01)
1.28 (1.18, 1.40)
1.18 (1.09, 1.27)
2.2 (1.4e−01)
0.98 (0.75, 1.28)
0.67 (0.58, 0.77)
6.4 (1.1e−02)
Smoker (ever vs never)
1.10 (0.98, 1.23)
0.99 (0.88, 1.12)
1.5 (2.1e−01)
1.09 (1.02, 1.17)
0.99 (0.90, 1.08)
3.3 (7.1e−02)
0.99 (0.81, 1.22)
0.94 (0.79, 1.12)
0.2 (6.8e−01)
Alcohol drinker (ever vs never)
1.11 (1.01, 1.22)
0.96 (0.83, 1.12)
2.4 (1.2e−01)
1.11 (1.05, 1.18)
0.98 (0.88, 1.09)
4.3 (3.9e−02)
1.06 (0.88, 1.27)
0.90 (0.72, 1.13)
1.1 (2.9e−01)
Physical activity (Q1 vs Q2−5)
1.19 (1.06, 1.34)
1.05 (0.98, 1.12)
3.5 (6.2e−02)
1.04 (0.96, 1.13)
0.97 (0.92, 1.02)
2.5 (1.1e−01)
1.05 (0.84, 1.31)
1.04 (0.94, 1.16)
0.0 (9.7e−01)
Fish consumption (<1 day/week vs weekly)
1.10 (0.98, 1.24)
1.07 (1.00, 1.15)
0.2 (6.5e−01)
1.09 (1.02, 1.17)
1.06 (1.01, 1.12)
0.4 (5.1e−01)
0.83 (0.67, 1.02)
1.00 (0.90, 1.11)
2.5 (1.1e−01)
Fresh fruit consumption (<1 day/week vs weekly)
1.05 (0.95, 1.15)
1.06 (1.00, 1.14)
0.1 (7.7e−01)
1.08 (1.02, 1.14)
1.07 (1.02, 1.12)
0.1 (7.8e−01)
0.93 (0.77, 1.13)
0.99 (0.90, 1.09)
0.3 (6.0e−01)
Medical history and health status
Self−rated poor health (yes vs no)
1.18 (1.01, 1.37)
1.23 (1.14, 1.33)
0.3 (6.0e−01)
1.02 (0.93, 1.13)
1.15 (1.09, 1.22)
4.4 (3.5e−02)
1.53 (1.17, 2.01)
1.51 (1.34, 1.69)
0.0 (9.0e−01)
Diabetes (yes vs no)
1.25 (1.05, 1.49)
1.31 (1.20, 1.44)
0.2 (6.3e−01)
1.18 (1.05, 1.33)
1.29 (1.21, 1.39)
1.8 (1.8e−01)
1.27 (0.91, 1.76)
1.03 (0.88, 1.20)
1.2 (2.6e−01)
Prior fracture (yes vs no)
1.63 (1.42, 1.86)
1.60 (1.46, 1.74)
0.1 (8.2e−01)
1.43 (1.32, 1.55)
1.52 (1.42, 1.62)
1.3 (2.5e−01)
1.35 (1.02, 1.79)
1.45 (1.26, 1.66)
0.2 (6.7e−01)
Prior rheumatoid arthritis (yes vs no)
1.10 (0.80, 1.50)
1.21 (1.05, 1.39)
0.3 (5.9e−01)
1.23 (1.01, 1.49)
1.18 (1.06, 1.31)
0.1 (7.2e−01)
2.60 (1.75, 3.88)
1.04 (0.83, 1.30)
15.5 (8.4e−05)
Prior CVD (yes vs no)
1.36 (1.13, 1.63)
1.20 (1.07, 1.34)
1.3 (2.5e−01)
1.28 (1.11, 1.46)
1.20 (1.10, 1.32)
0.5 (4.9e−01)
1.23 (0.88, 1.70)
1.13 (0.96, 1.34)
0.2 (6.7e−01)
Prior cancer (yes vs no)
1.43 (0.81, 2.54)
1.16 (0.82, 1.63)
0.4 (5.3e−01)
1.43 (0.97, 2.10)
0.96 (0.72, 1.28)
2.6 (1.1e−01)
2.38 (1.06, 5.36)
0.82 (0.45, 1.49)
4.3 (3.8e−02)
Hypertension (yes vs no)
0.99 (0.90, 1.09)
0.92 (0.86, 0.97)
1.9 (1.6e−01)
0.93 (0.88, 0.99)
0.92 (0.88, 0.96)
0.2 (6.4e−01)
0.80 (0.67, 0.97)
0.88 (0.80, 0.96)
0.7 (3.9e−01)
Anthropometry
Standing height (Q5 vs Q1−4)
1.17 (1.02, 1.34)
1.10 (1.01, 1.20)
0.5 (4.9e−01)
1.07 (0.99, 1.16)
1.03 (0.97, 1.10)
0.4 (5.5e−01)
1.10 (0.85, 1.43)
1.01 (0.88, 1.16)
0.3 (5.6e−01)
Weight (Q1 vs Q2−5)
1.22 (1.08, 1.36)
1.04 (0.96, 1.11)
5.4 (2.0e−02)
1.11 (1.04, 1.19)
0.99 (0.95, 1.04)
6.7 (9.6e−03)
0.83 (0.66, 1.05)
1.02 (0.92, 1.14)
2.5 (1.1e−01)
Waist−hip ratio (Q1 vs Q2−5)
0.97 (0.87, 1.09)
0.86 (0.79, 0.93)
3.2 (7.4e−02)
1.01 (0.95, 1.08)
0.90 (0.85, 0.95)
7.3 (6.8e−03)
0.97 (0.78, 1.20)
0.85 (0.76, 0.96)
1.1 (3.0e−01)
Men
Women
0.5 1 2 4
Hazard Ratio (95% CI)
0.5 1 2 4
Hazard Ratio (95% CI)
0.5 1 2 4
Hazard Ratio (95% CI)

eFigure 5. Associations of risk factors with hip fracture in men and women, after excluding participants with history of fracture

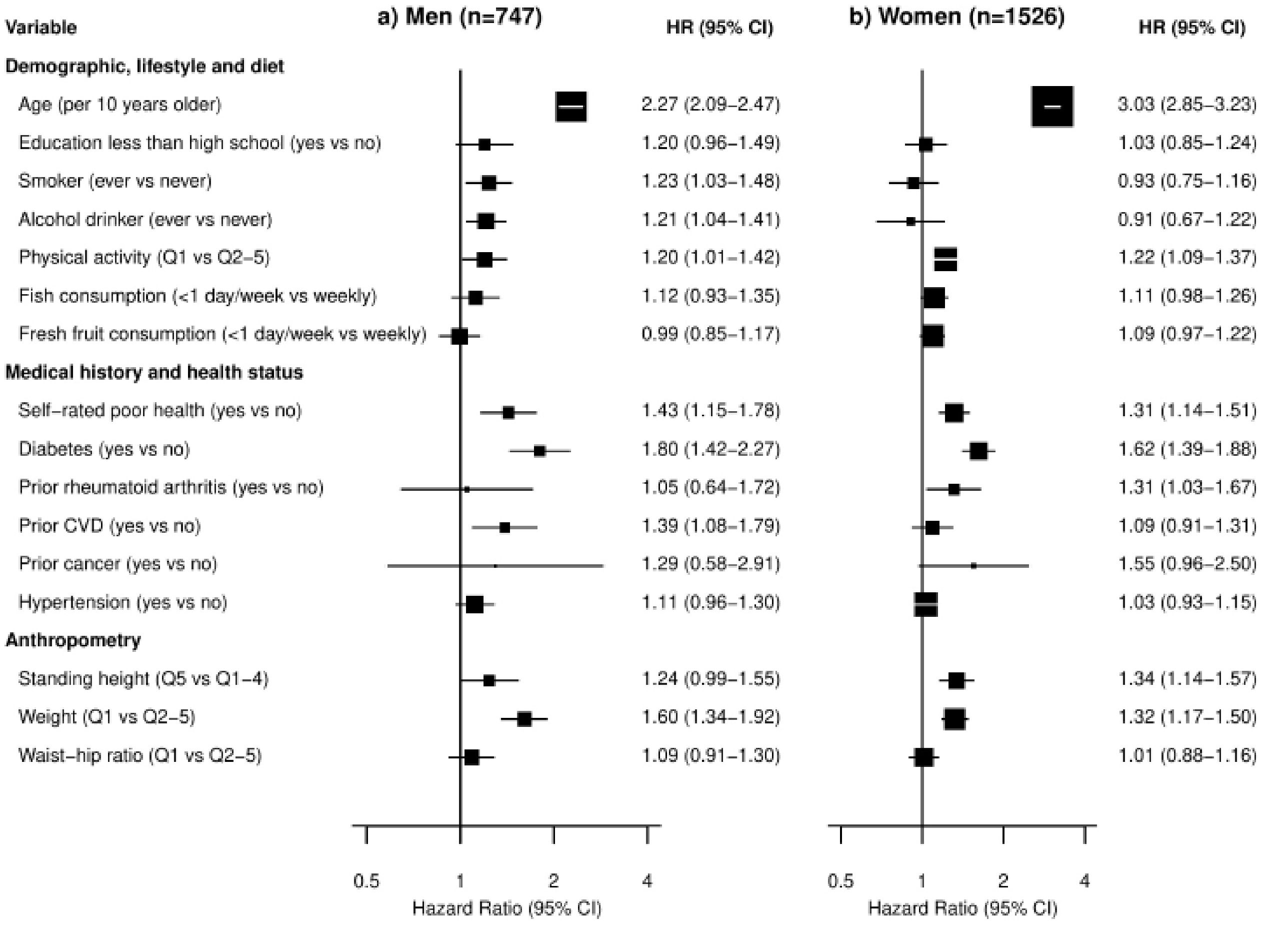

eFigure 6. Associations of risk factors with hip fracture in men and women, after excluding the first 5 years of follow-up

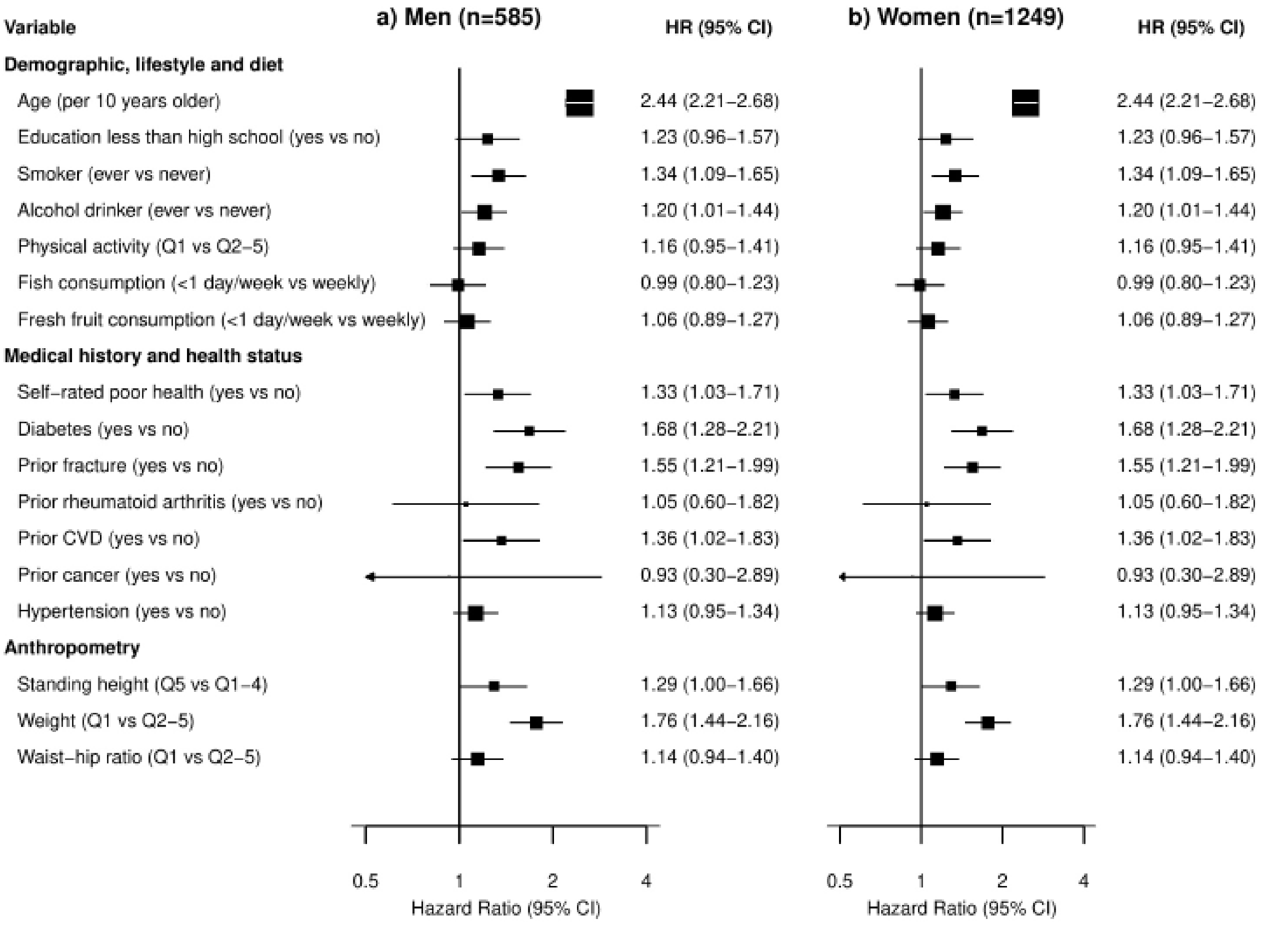

eFigure 7. Associations of risk factors with hip fracture in men and women, after excluding participants with history of fracture, and the first 5 years of follow-up

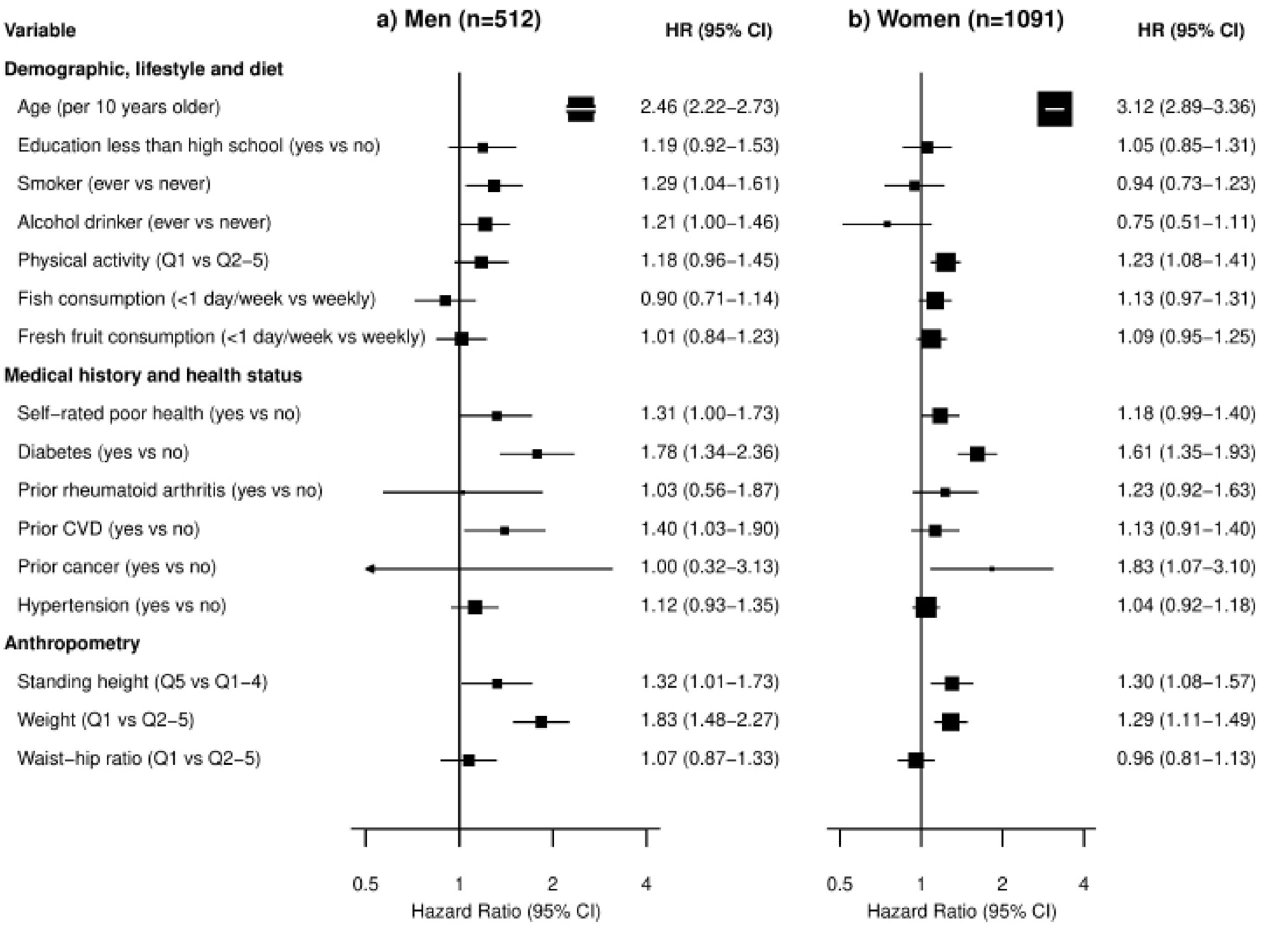

eFigure 8. Age- and sex-specific incidence rates of fractures in CKB and UK

Fracture incidence data are from the Clinical Practice Research Datalink (CPRD), which contains anonymised electronic health records for approximately 6.9% of the UK population. Information comes from General Practitioners, and covers 11.3 million people from 674 practices across the UK, demonstrated to be representative of the national population (*Curtis, et al, Bone. 2016;87:19-26*).

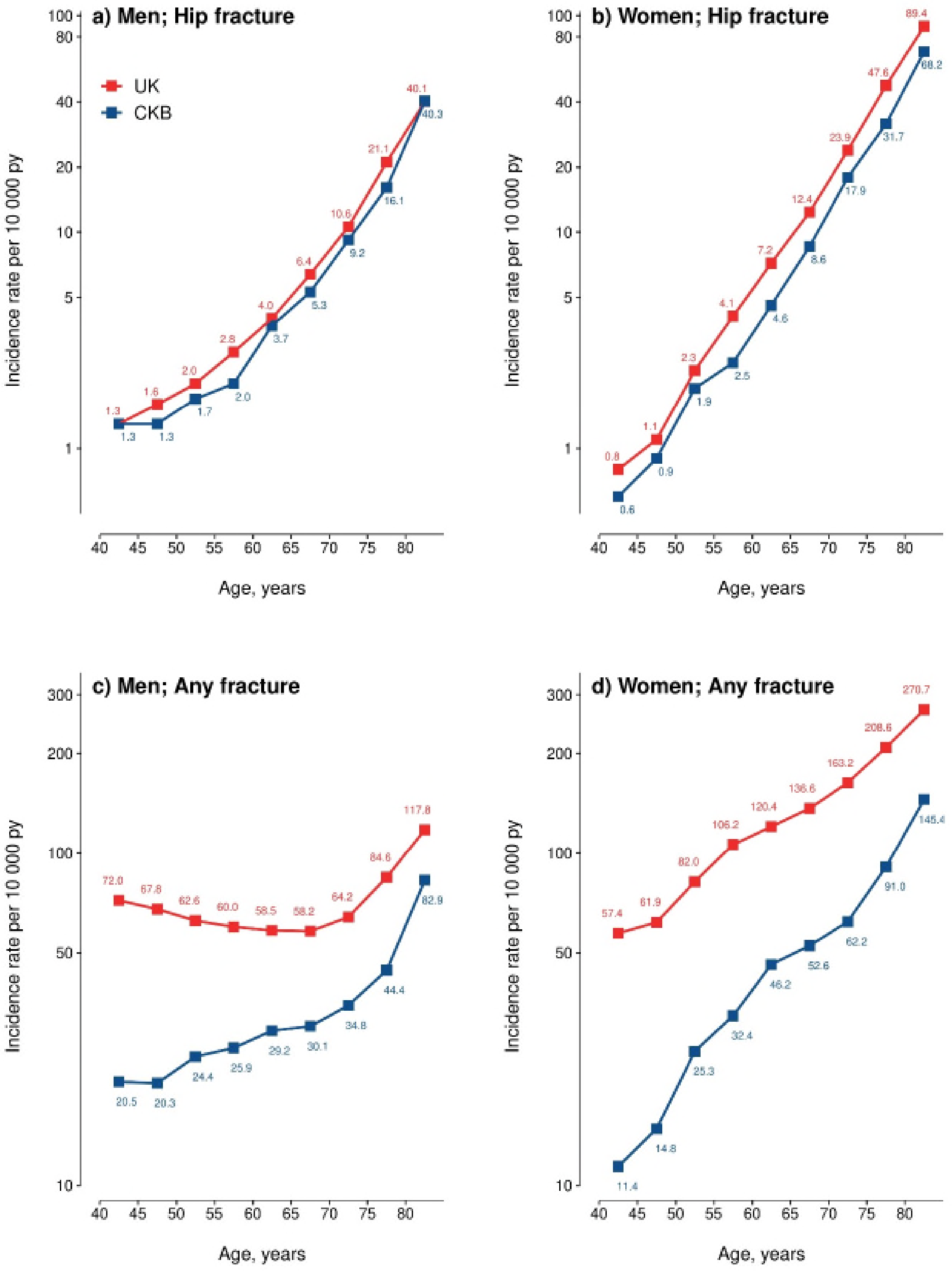

Supplement: Supplementary file 1 [file EMS141051-supplement-supplementary.pdf]
